# Supplementary material for: Targeting N-myristoylation for therapy of B-cell lymphomas
Source: Nat Commun. 2020 Oct 22;11:5348. doi: 10.1038/s41467-020-18998-1 (PMC7582192; doi:10.1038/s41467-020-18998-1)
Supplement: Supplementary file 1 — Supplementary Information [file 41467_2020_18998_MOESM1_ESM.pdf]

SUPPLEMENTARY INFORMATION

Targeting N-myristoylation for therapy of B-cell lymphomas by Beauchamp et al.

SUPPLEMENTARY FIGURES

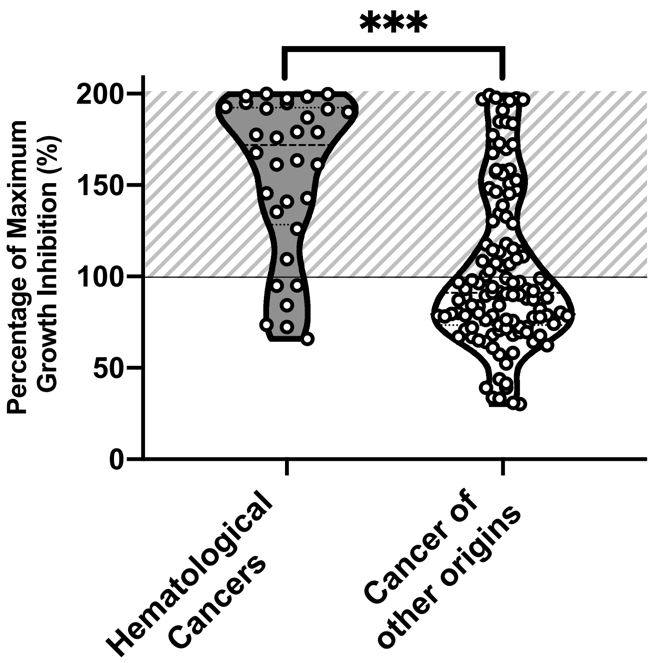

Supplementary Figure 1. Combined Horizon and Oncoline cell line screen data demonstrates that PCLX-001 confers maximal growth inhibition on hematologic cancer cell lines in comparison to cell lines derived from all other cancer types. Violin graph depicting the combined percentage growth inhibition of PCLX-001on hematological cell lines versus all other non-hematological cell lines from both the Horizon and Oncoline cell line screens following 96hrs of treatment. Quartiles are separated by dotted lines. (***) indicates a significant difference in growth inhibition (Unpaired t-test, two-tailed *P*<0.0001).

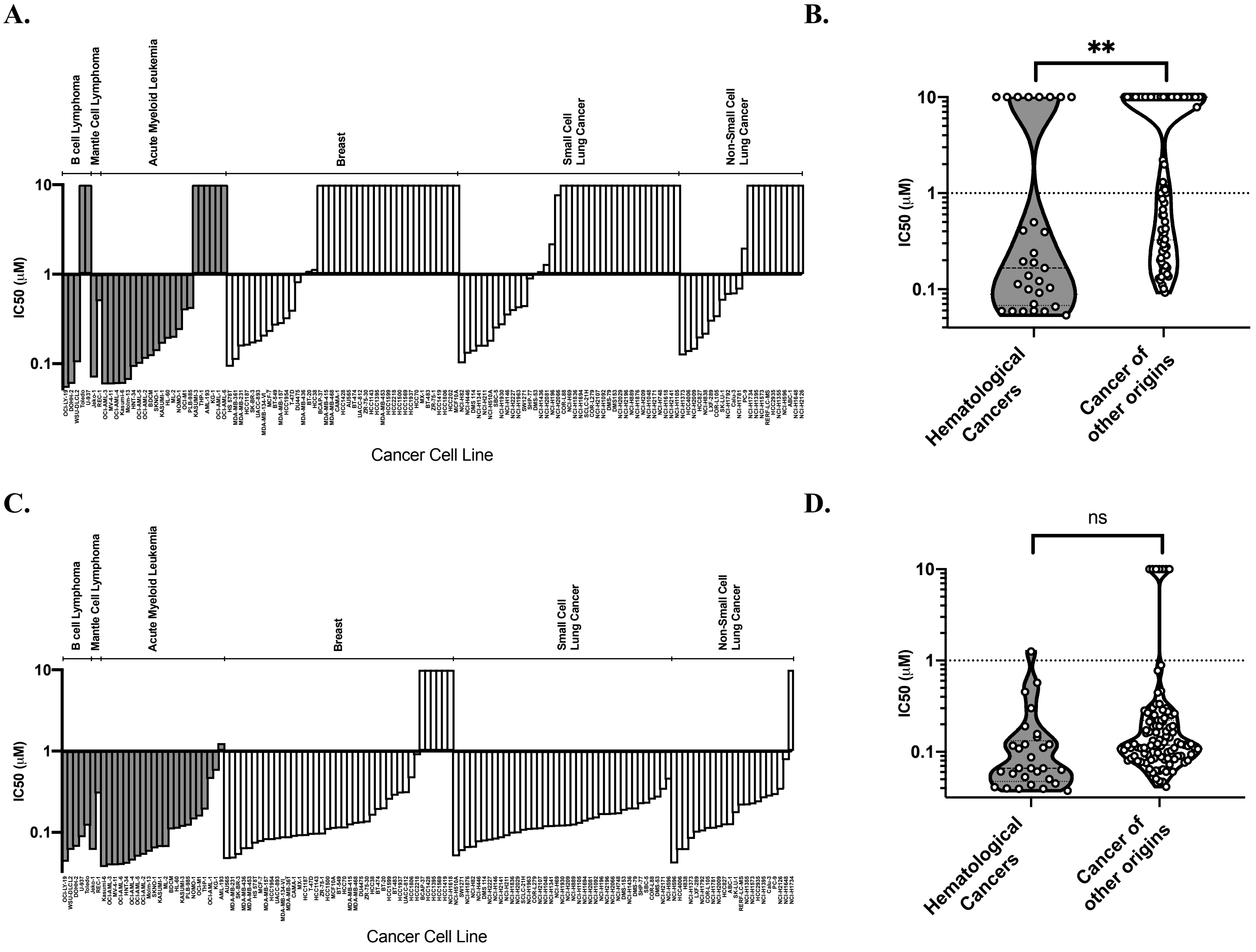

Supplementary Figure 2. Breadth of efficacy screen demonstrates that PCLX-001 is active against various other cancer cell lines including those derived from solid tumors. Absolute IC_{50} values of various cell lines treated for 3 days (A, B) or 6 days (C, D) with 0.0005 - 10µM PCLX-001. Cell lines are arranged according cancer type. Individual bars represent a single cancer cell line derived from B cell lymphoma and Mantle Cell Lymphoma, Acute Myeloid Leukemia (AML), Breast, Small-cell lung carcinoma (SCLC), Non-small-cell lung carcinoma (NSCLC). ChemPartner robotic platform determined cell viability using CellTiter Blue viability assay. Growth inhibition (GI) was not calculated since the viability of the cells at Day 0 was not available from the Chempartner platform. (Unpaired t-test, two-tailed, ** P=0.0038, ns= non significant).

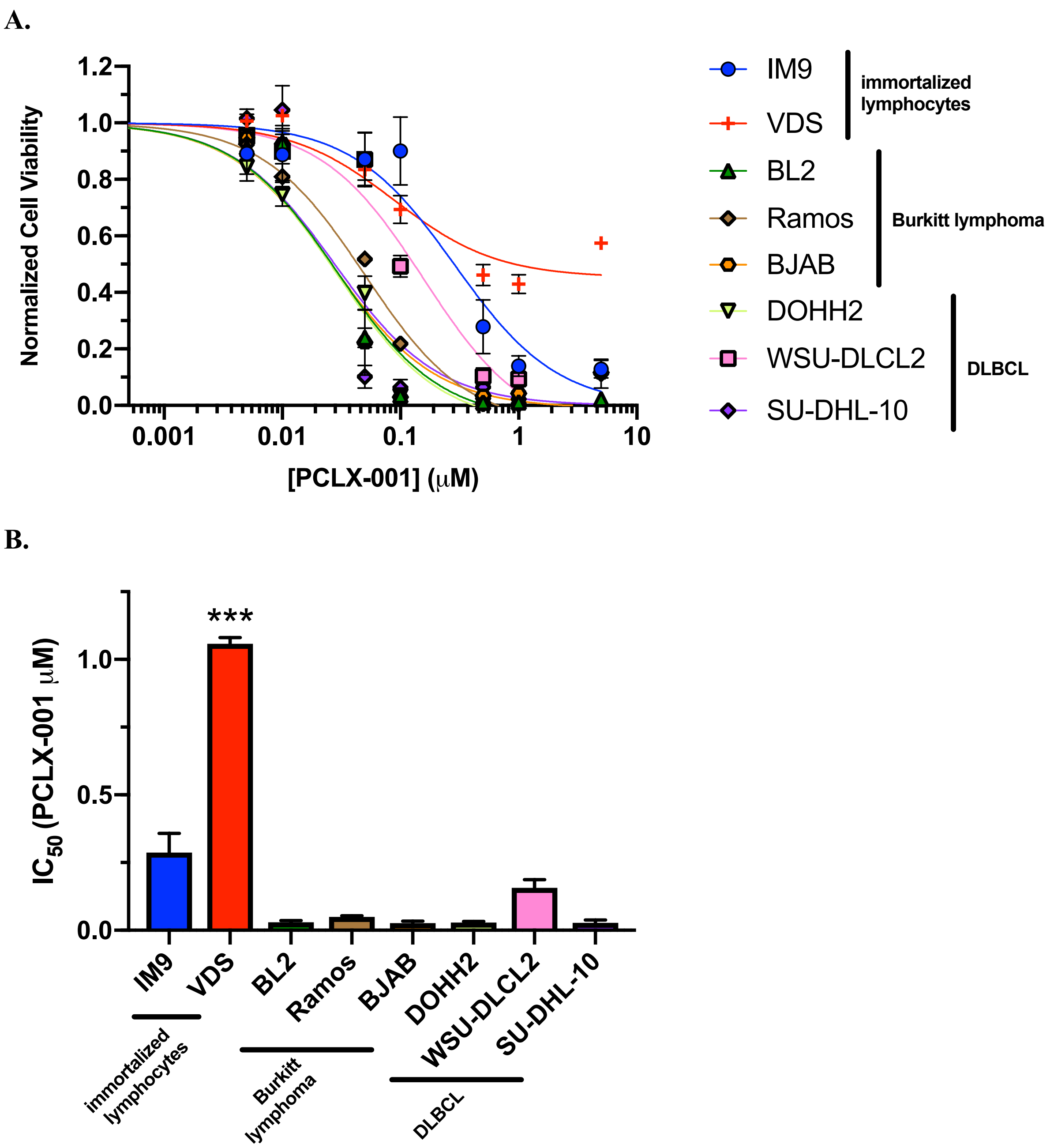

Supplementary Figure 3. PCLX-001 selectively kills hematological cancer cell lines in comparison to immortalized lymphocytes. (A) Normalized cell viability curves of immortalized lymphocytes (IM9, VDS), BL (BL2, Ramos, BJAB), and DLBCL (DOHH2, WSU-DLCL2, SU-DHL-10) cell lines treated with 0.001 - 5μM of PCLX-001 for 96hrs, as determined by Calcein Assay, which measures the percentage of viable cells regardless of the number of cells. (B) Corresponding histograms of absolute IC_{50} (and SD) values calculated from a log(inhibitor) vs response (3 parameters) equation from cell viability curves plotted in (A). Values are mean ± s.e.m. of 3 experiments. (Ordinary one-way Anova, Tukey's multiple comparisons test, *** *P*<0.0001).

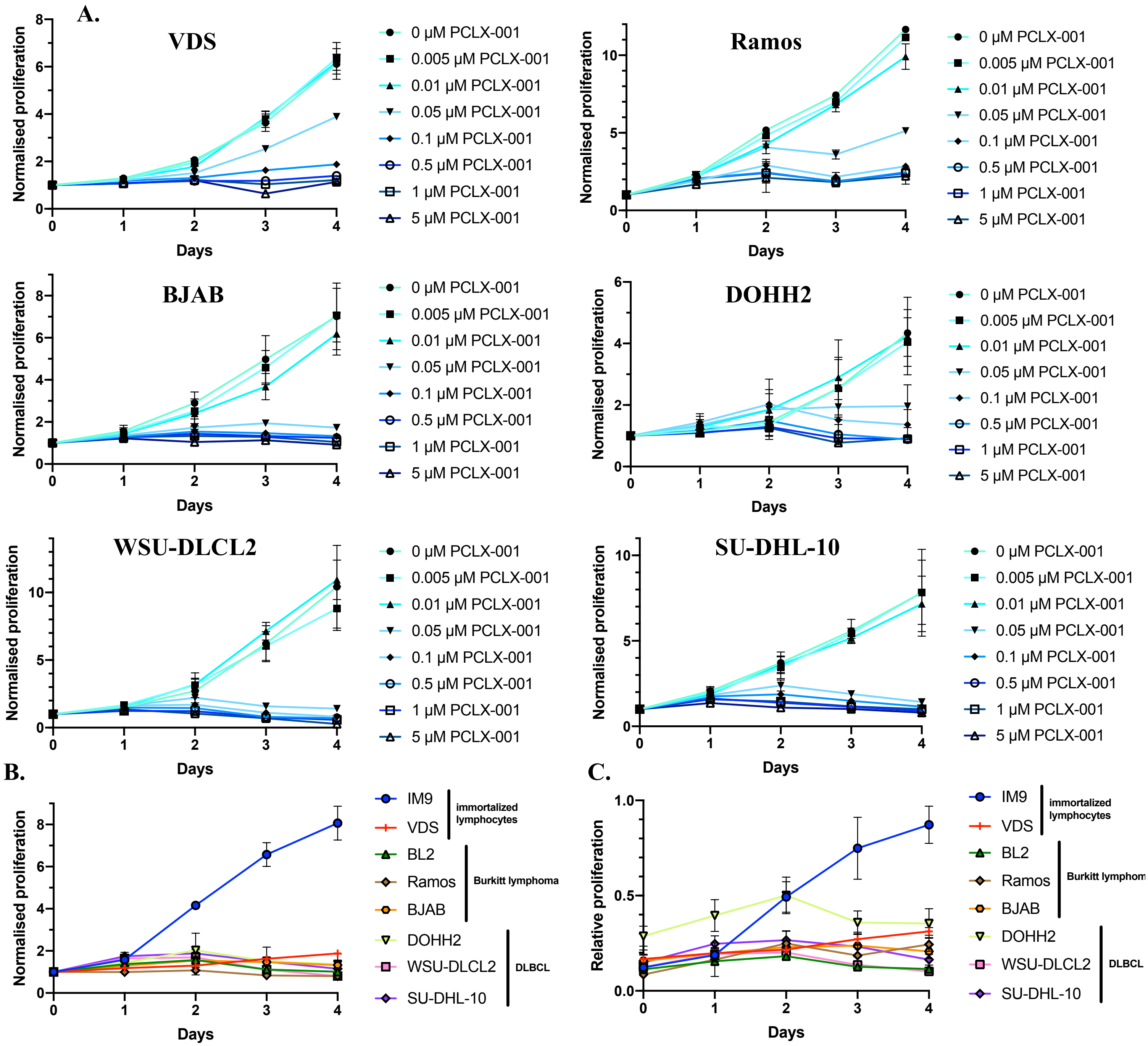

Supplementary Figure 4. PCLX-001 treatment decreases the normalized lymphoma cell line proliferation. (A) Normalized proliferation of immortalized lymphocyte (VDS), BL (Ramos, BJAB), and DLBCL (DOHH2, WSU-DLCL2, SU-DHL-10) cell lines treated with 0 - 5 µM of PCLX-001 for 96hrs as determined by cell count. (B) Inhibition of the normalized proliferation of various cell lines after 0.1µM PCLX-001 treatment up to 96hrs. (C) To account for the differences in cell growth rates were transformed our data into a relative ratio of the normalized proliferation of various cell lines after 0.1µM PCLX-001 treatment up to 96hrs divided by the normalized proliferation of the respective untreated cell lines. Values are mean ± s.e.m. of 3 experiments.

A.

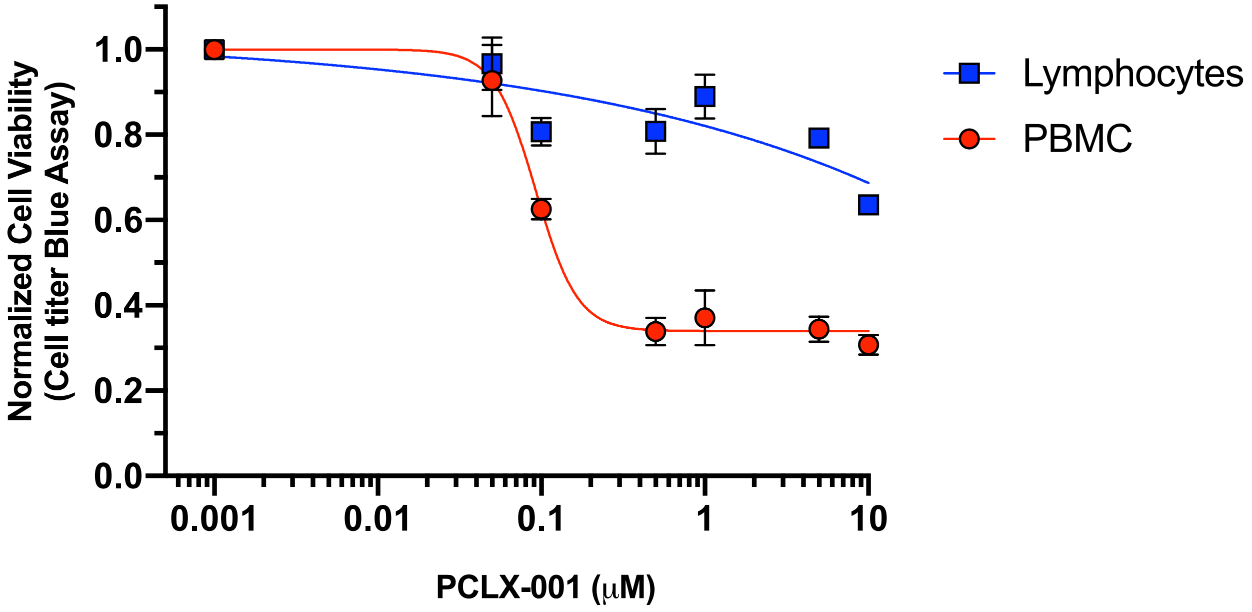

B.

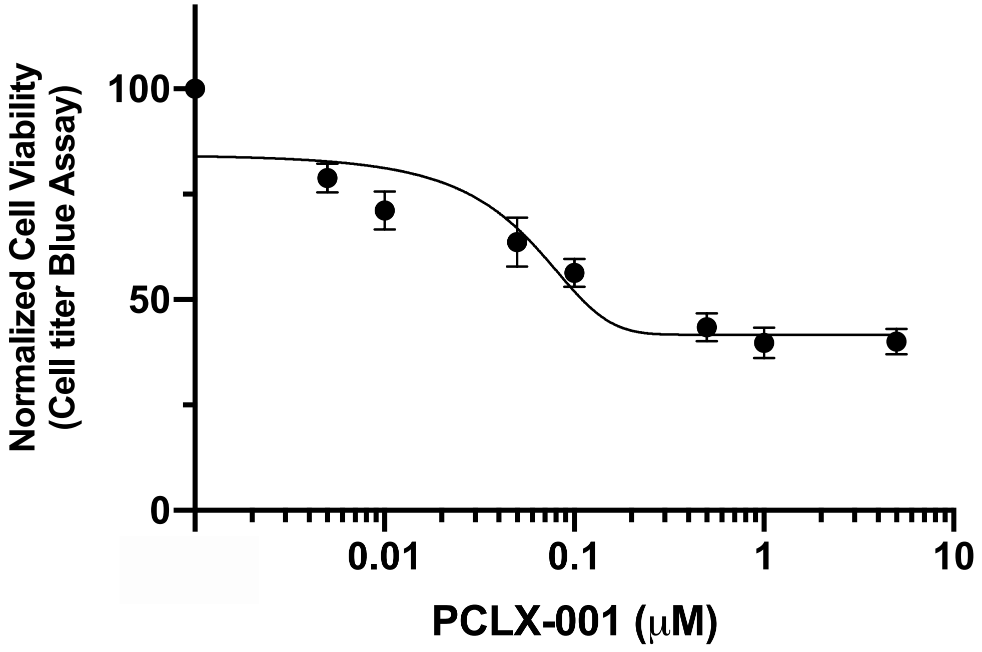

Supplementary Figure 5. A large proportion of freshly isolated human lymphocytes, PBMCs and primary umbilical vein endothelial cells (HUVEC) are resistant to PCLX-001. Cell viability curve of 2 freshly isolated human peripheral blood monocytes (PBMC) and lymphocytes preparations treated for 96hrs with 0.001 - 10μM PCLX-001. Values are mean ± s.e.m. (n=2). HUVECs were treated for 96hrs with 0.001 - 5μM PCLX-001 and residual cell viability was determined using a Cell-Titer Blue Assay. Values are mean ± S.D. (n=4).

A.

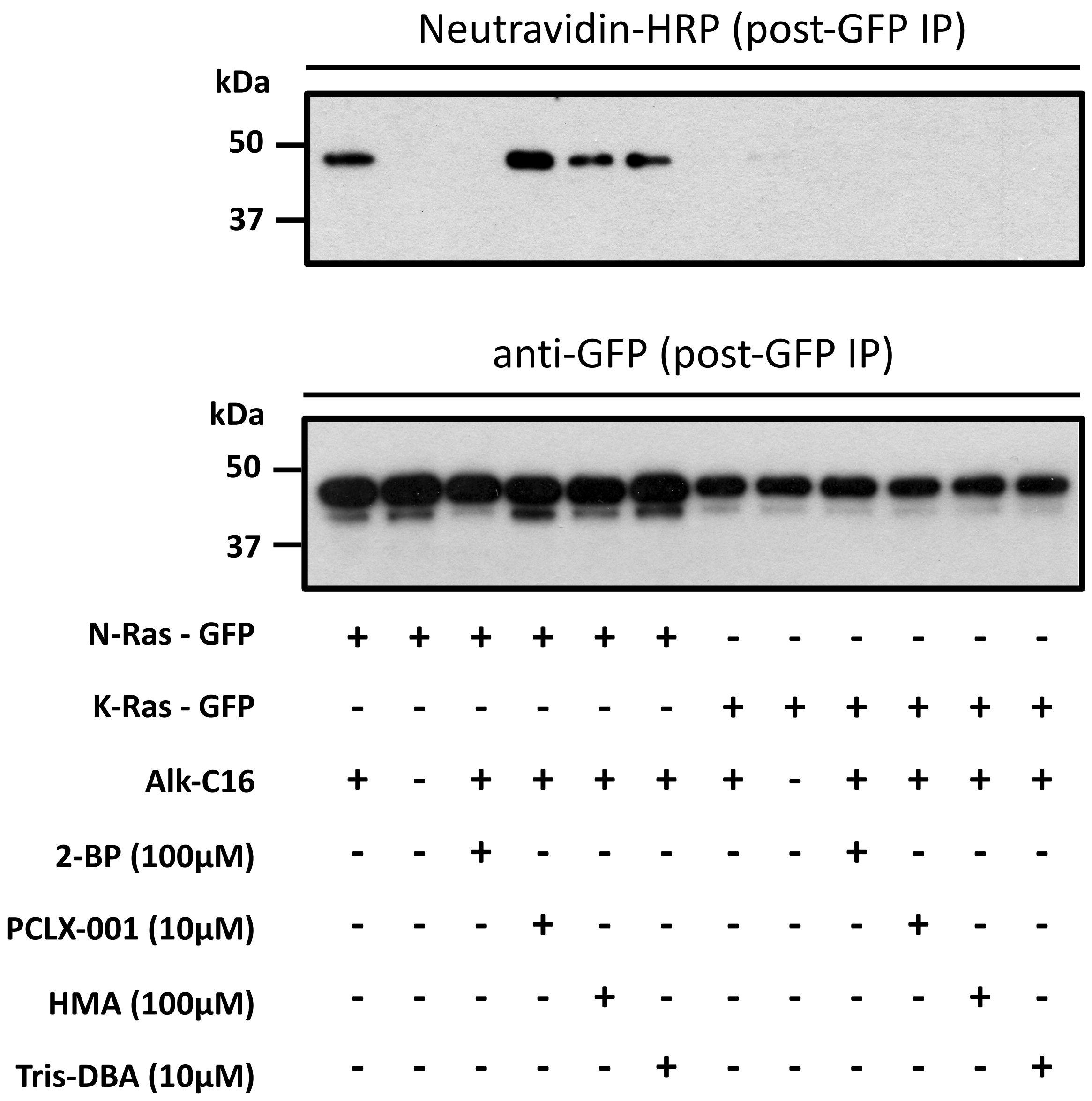
Neutravidin-HRP (post-GFP IP)
kDa
50
37
anti-GFP (post-GFP IP)
kDa
50
37
N-Ras - GFP + + + + + + - - - - - -
K-Ras - GFP - - - - - - + + + + + +
Alk-C16 + - + + + + + - + + + +
2-BP (100μM) - - + - - - - - + - - -
PCLX-001 (10μM) - - - + - - - - - + - -
HMA (100μM) - - - - + - - - - - + -
Tris-DBA (10μM) - - - - - + - - - - - +

B.

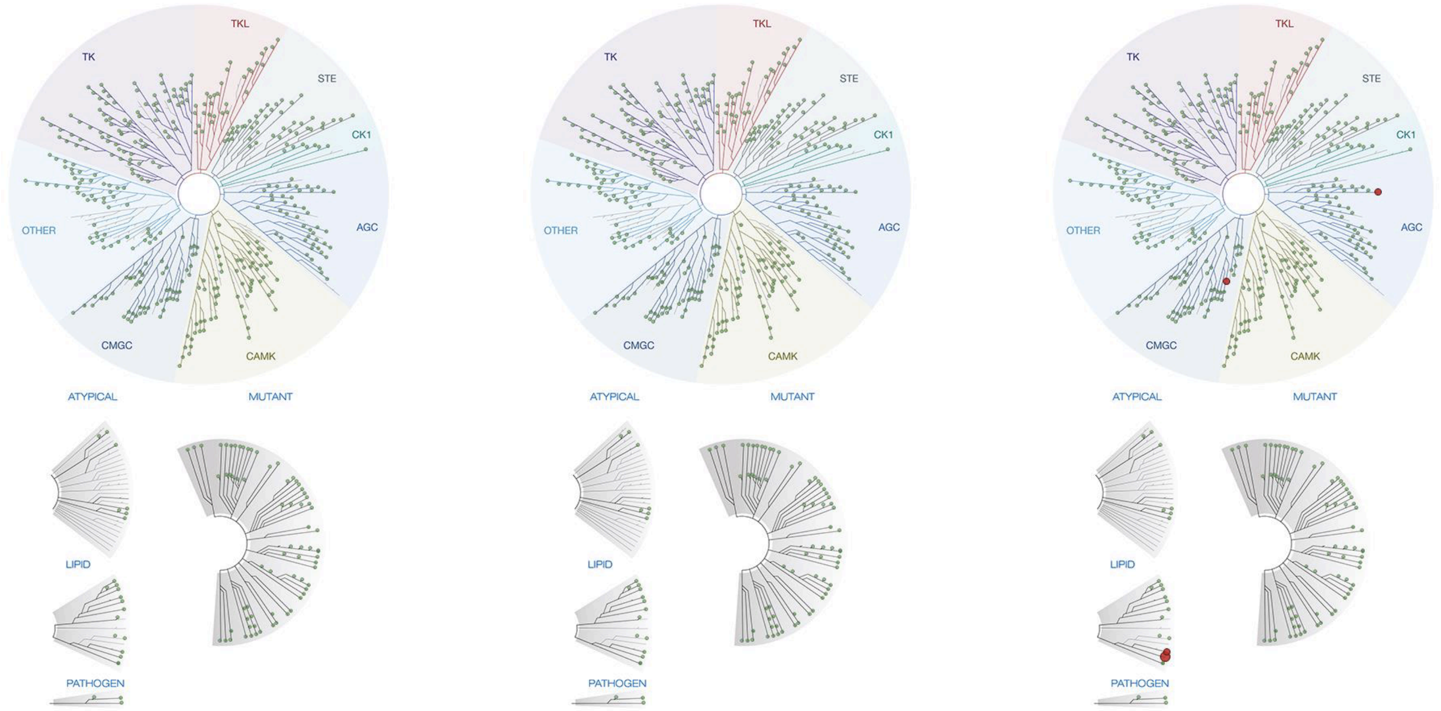

PCLX-001 (0.5µM) | **PCLX-001 (10µM)** | **PCLX-001 (100µM)**

Supplementary Figure 6. PCLX-001 does not inhibit palmitoylation of Ras and does not have any significant off-target kinase inhibitor activity at physiological level. (A) COS-7 cells transiently expressing palmitoylatable EGFP-N-Ras or non-palmitoylatable EGFP-K-Ras for 48hrs were pre-treated for 1hr with 100µM 2-bromopalmitate (2-BP), a palmitoylation inhibitor or the following NMT inhibitors: 10µM PCLX-001, 100µM 2-hydroxymyristate (HMA) or 10µM Tris-DBA. The cells pre-treated with inhibitors were then labelled for 4hrs with 100µM Alkyne-C16. EGFP-tagged constructs were immunoprecipitated as described and reacted with azido-biotin using click chemistry. Biotinylated-palmitoylated proteins were detected using neutravidin-HRP conjugate and ECL. (B) TREEspot™ is a proprietary data visualization software tool developed by DiscoverX Corporation, CA, USA. 468 pre-configured human kinases of the scanMAX KINOMEscan were tested. Mutant and lipid kinases are not represented. Possible kinases found to bind PCLX-001 are marked with red circles, where larger circles indicate higher-affinity binding. No kinases were found binding with PCLX-001 up to 10µM, which corresponds to a ~400 times larger concentration than the PCLX-001 EC_{50} for BL2 cells . At 100µM PCLX-001 (~4000 times the EC_{50} for BL2), only 3 kinases (MRCKA, PIP5K2C and SRPK1 shown in red) were found to weakly bind PCLX-001 (kinase activity score < 35% of control). All western blots shown are representative of three independent experiments.

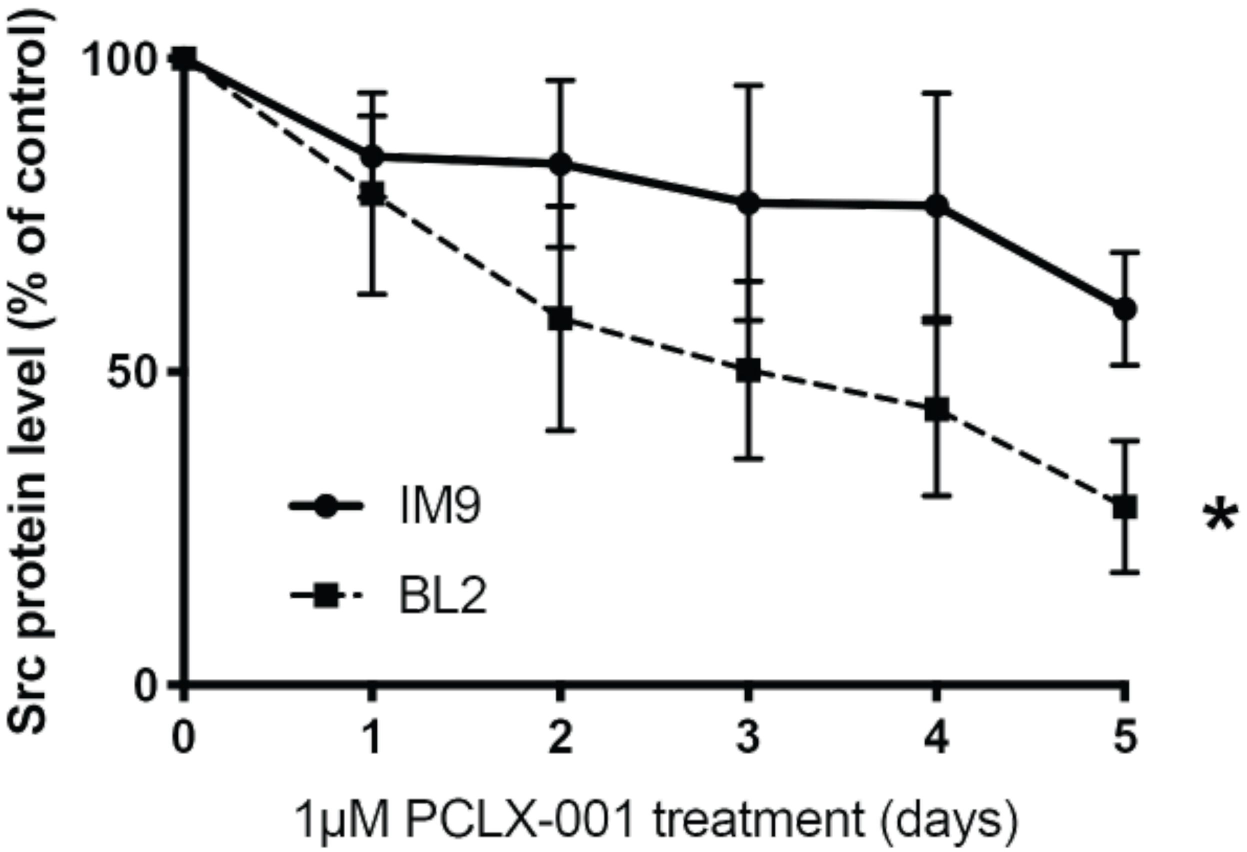

Supplementary Figure 7. Quantification of the Src protein level decrease in BL2 and IM9 cells treated with PCLX-001 for up to 5 days. Quantification of total endogenous Src protein levels detected by Western blot (Fig. 2F). Errors bars depict standard error from the mean. (*) indicates a significant difference (2way ANOVA, P=0.0174) in Src protein levels (n=3).

A.

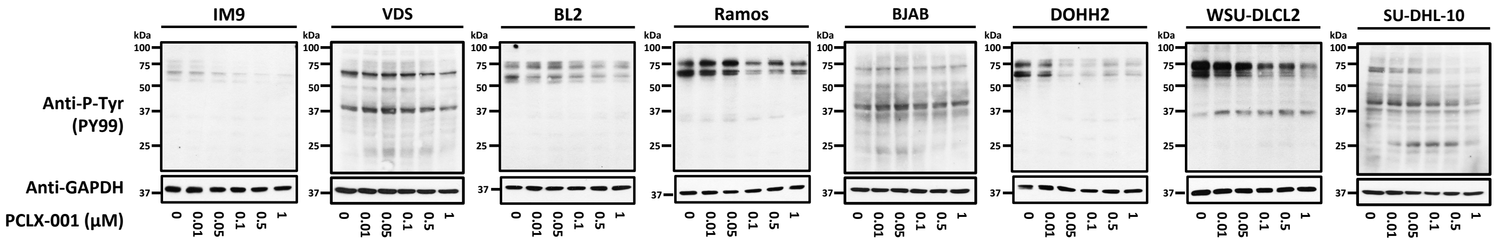

B.

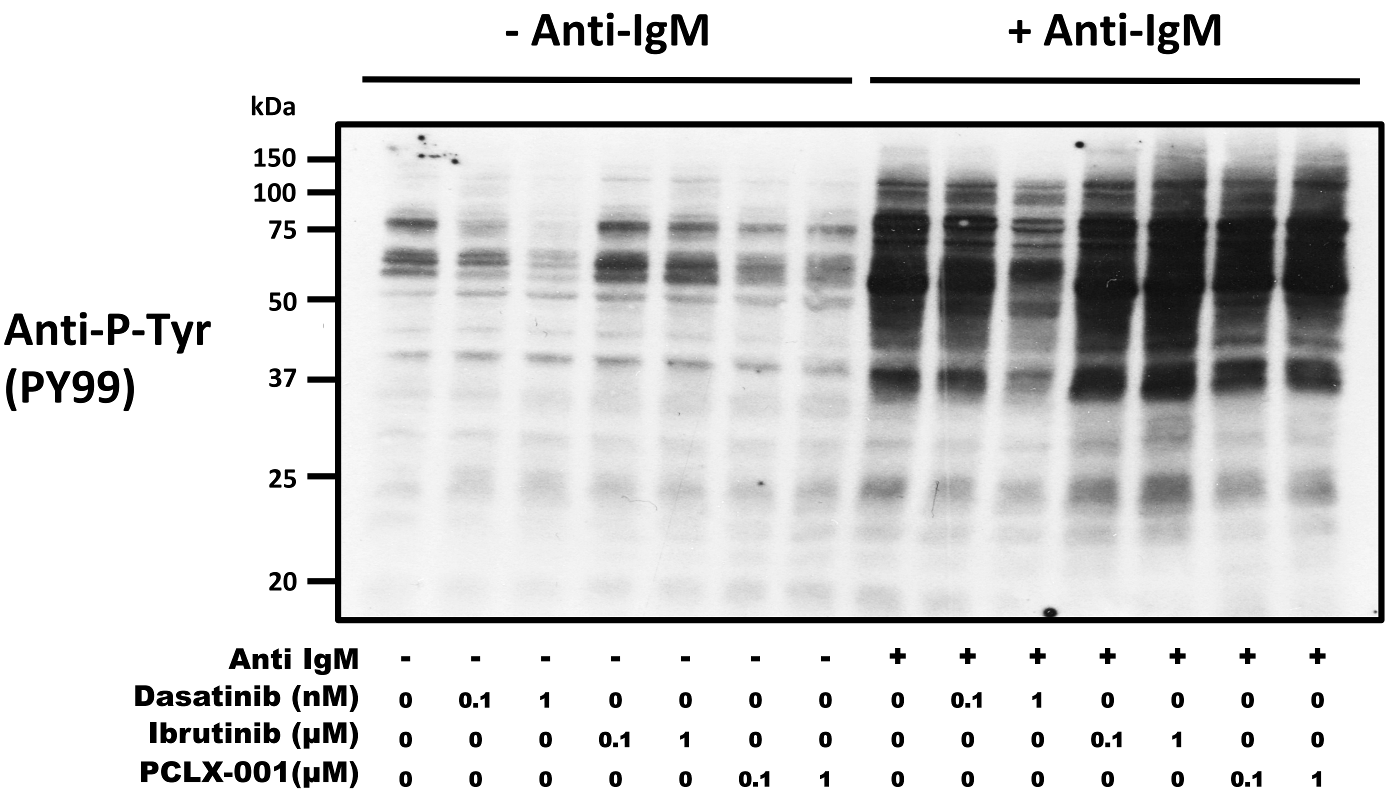

Supplementary Figure 8. PCLX-001 treatment reduces phospho-tyrosine levels in basal (tonic or chronic) and anti-IgM activated signaling in various normal and malignant B cell lines. (A) Western blots assessing the basal (antigen independent tonic or chronic) tyrosine phosphorylation levels (PY99) in normal IM9 and VDS cell lines,and malignant B cell lines BL2, Ramos, BJAB, DOHH2, WSU-DLCL2 and SU-DHL-10 cells following 24hrs treatment with 0.01-1μM PCLX-001. (B) **PY99** Western blot of unstimulated (left) and anti-IgM ligated BCR (right) BL2 cells treated for 24hrs with 0.1μM or 1μM of dasatinib, ibrutinib or PCLX-001. BL2 cells were activated as indicated with 25μg/ml goat anti-human IgM for 2min. Western blots shown are representative of at least 3 independent experiments.

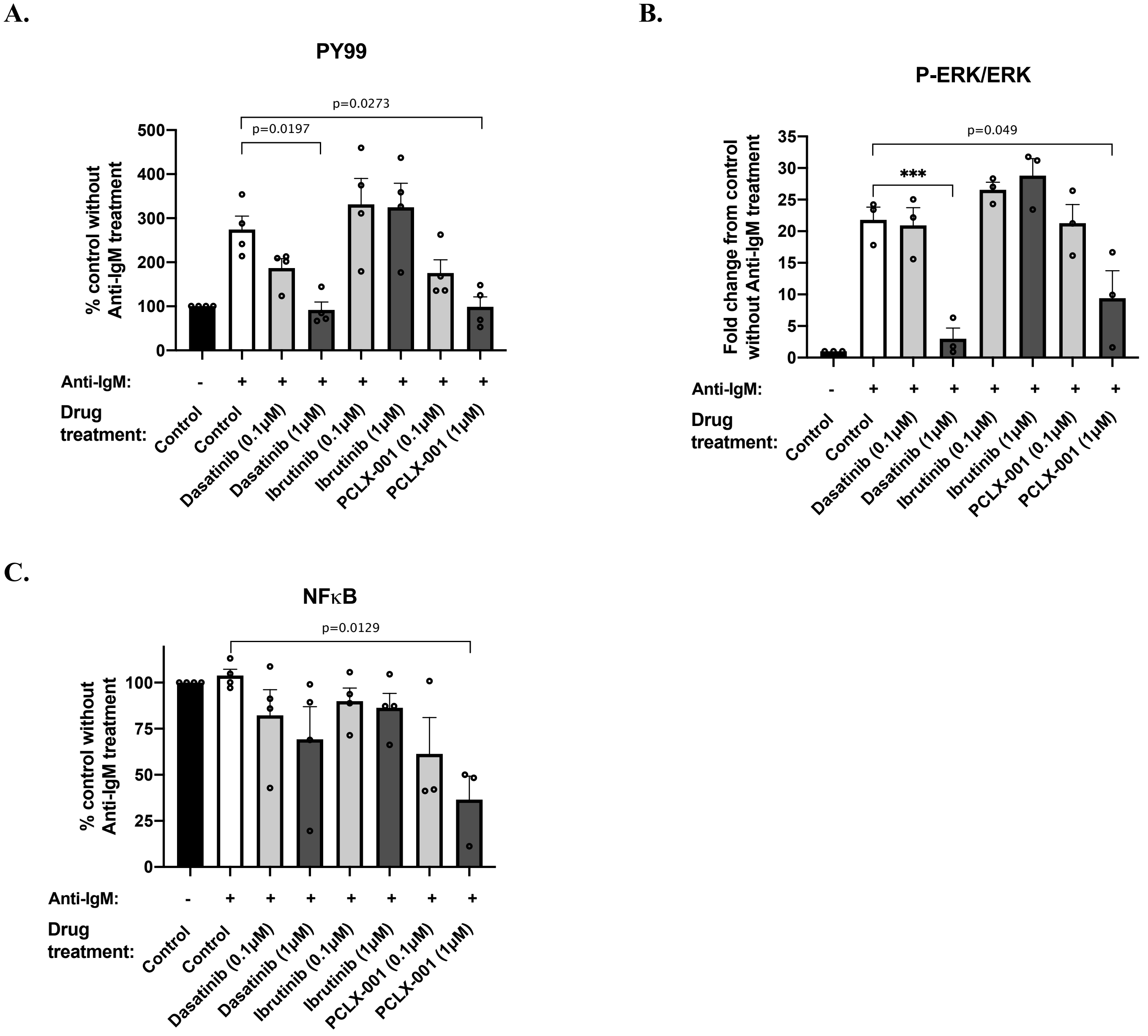

Supplementary Figure 9. PCLX-001 treatment significantly decreases total phospho-tyrosine, phospho-ERK (P-ERK) and NFκB levels in BL2 cells. Quantification of western blots for total phospho-tyrosine levels using PY99 antibody (A), P-ERK (B) and NFκB (C) in BL2 cells treated for 48hrs with 0.1µM or 1.0µM of dasatinib, ibrutinib or PCLX-001 (Fig 4A) (n=4 for A and C, n=3 for B). BL2 cells were activated with 25µg/ml goat anti-human IgM for 2min where indicated. Errors bars depict standard error from the mean. (*) indicates a significant difference (P<0.05) in phospho-tyrosine levels (Ordinary one-way Anova, Tukey's multiple comparisons test).

A.

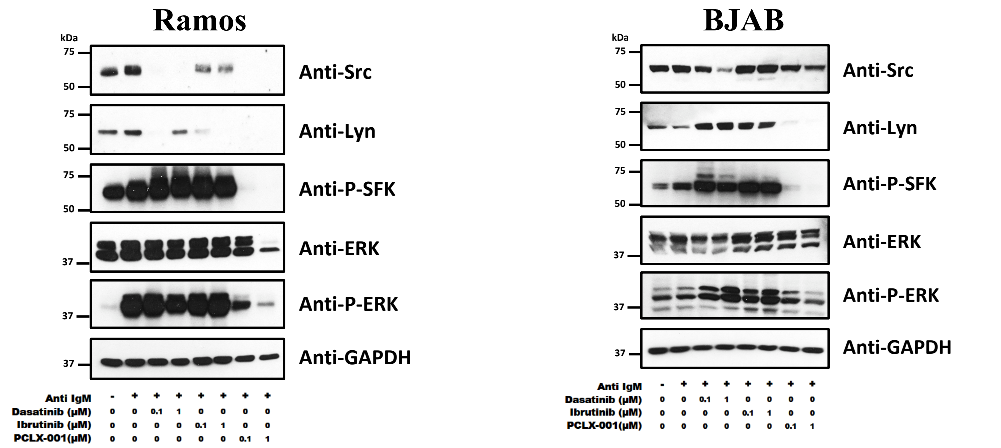

B.

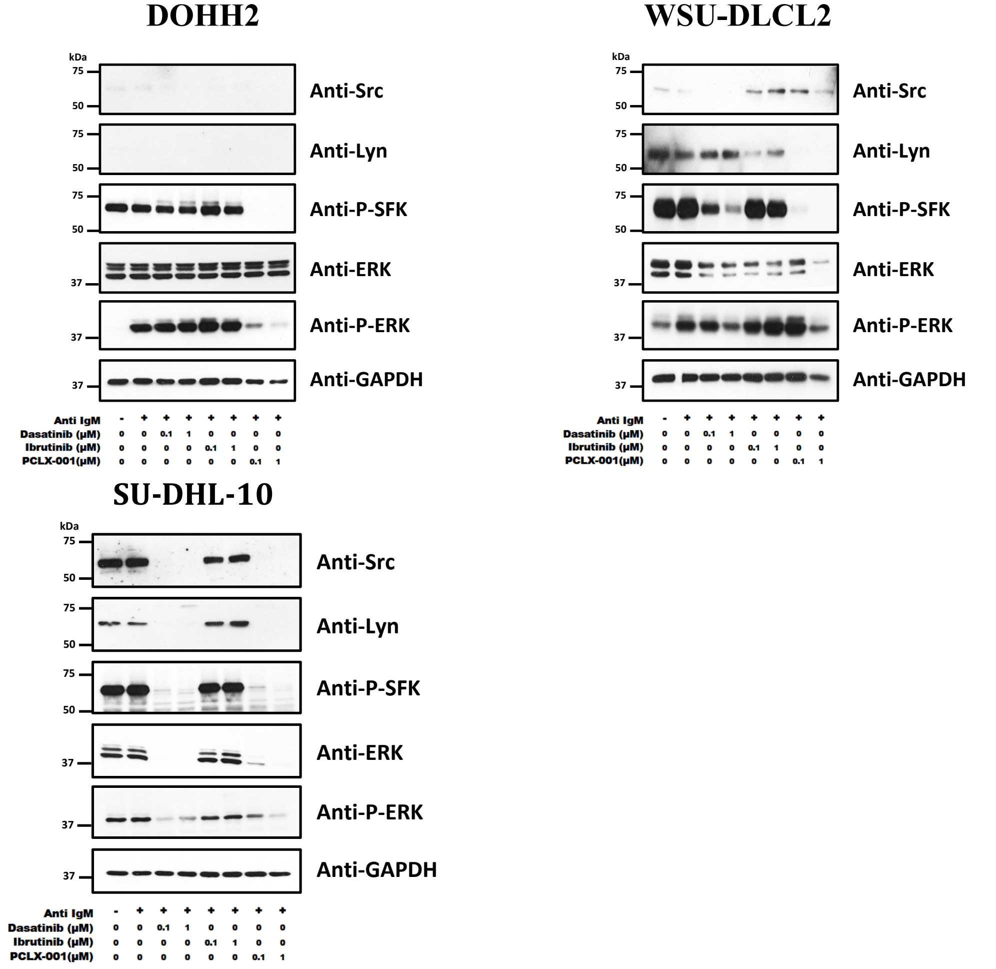

Supplementary Figure 10. PCLX-001 treatment attenuates anti-IgM ligated BCR signaling in various lymphoma cell lines. Western blots of (A) BL (Ramos, BJAB), and (B) DLBCL (DOHH2, WSU-DLCL2, SU-DHL-10) cell lines treated for 48hrs with 0.1µM or 1.0µM of dasatinib, ibrutinib or PCLX-001 to detect total Src, Lyn, ERK, phosphorylated SFKs (P-SFK) and phosphorylated ERK (P-ERK) levels. Src and Lyn were not detected in DOHH2. Western blots are representative of at least three independent experiments. GAPDH serves as a loading control. Cell lines were activated with 25µg/mL goat anti-human IgM for 2min prior to Western blotting. All western blots shown are representative of three independent experiments.

A.

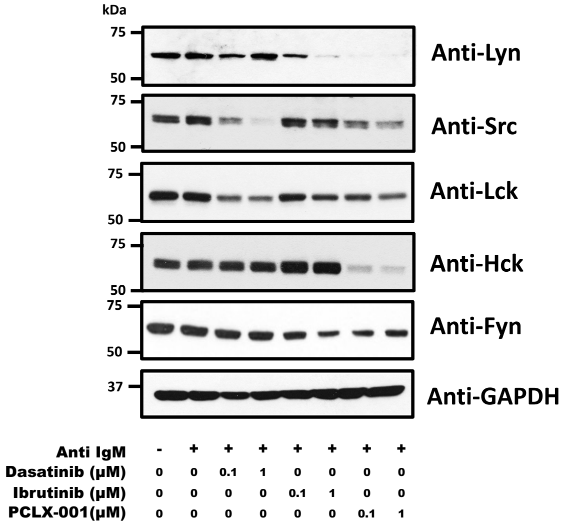

B.

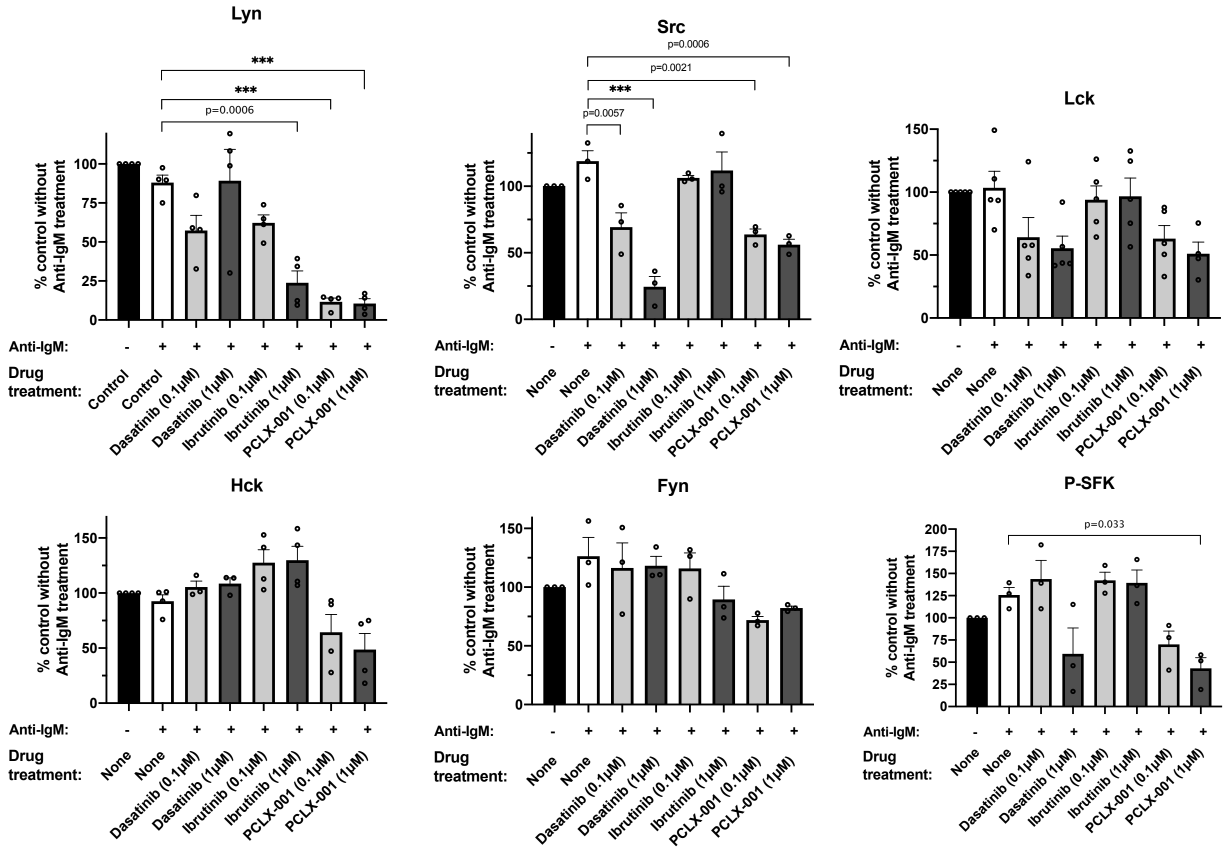

Supplementary Figure 11. Comparison of various SFK levels in BL2 cells following 48hr treatment with PCLX-001, dasatinib, ibrutinib. Western blot (A) and quantification (B) of the protein levels of Lyn, Src, Lck, Hck, Fyn, and total phosphorylated SFKs (P-SFK blot is shown in Fig. 4A) in BL2 cells treated for 48hrs with 0.1µM or 1.0µM of dasatinib, ibrutinib or PCLX-001. BL2 cells were activated with 25µg/ml goat anti-human IgM for 2min where indicated. Errors bars depict standard error from the mean. (***) indicates a significant difference ($p<0.0001$) in protein or phosphorylated protein levels (Ordinary one-way ANOVA, Tukey's multiple comparisons test).

A.

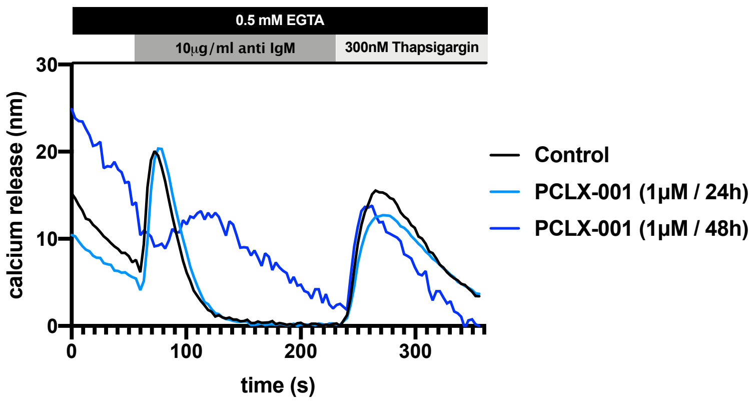

B.

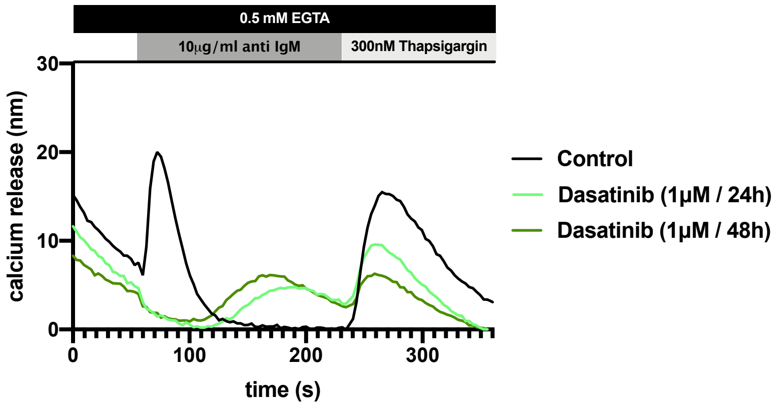

C.

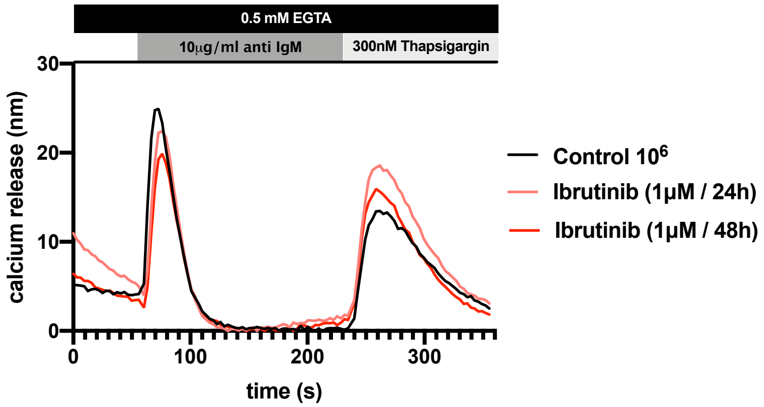

Supplementary Figure 12. PCLX-001 reduces BCR receptor-dependent calcium release activated by anti-IgM stimulation in BL2 cells. Endoplasmic reticulum Ca^{++} release was measured in BL2 cells treated with 1µM PCLX-001(A), Dasatinib (B) or Ibrutinib (C) for 24h or 48h. Following cell loading with the fluorescent Ca^{++} indicator Fura-2 cells were stimulated with 10µg/ml Goat F(ab')2 anti-human IgM to ligate and activate BCR-receptor dependent Ca^{++} release then following thapsigargin (300nM) treatment to show BCR-receptor independent Ca^{++} release from endoplasmic reticulum. Results shown are representative of multiple replicates of the experiment (n=6 for PCLX-001 incubation, n=3 for dasatinib and ibrutinib).

A.

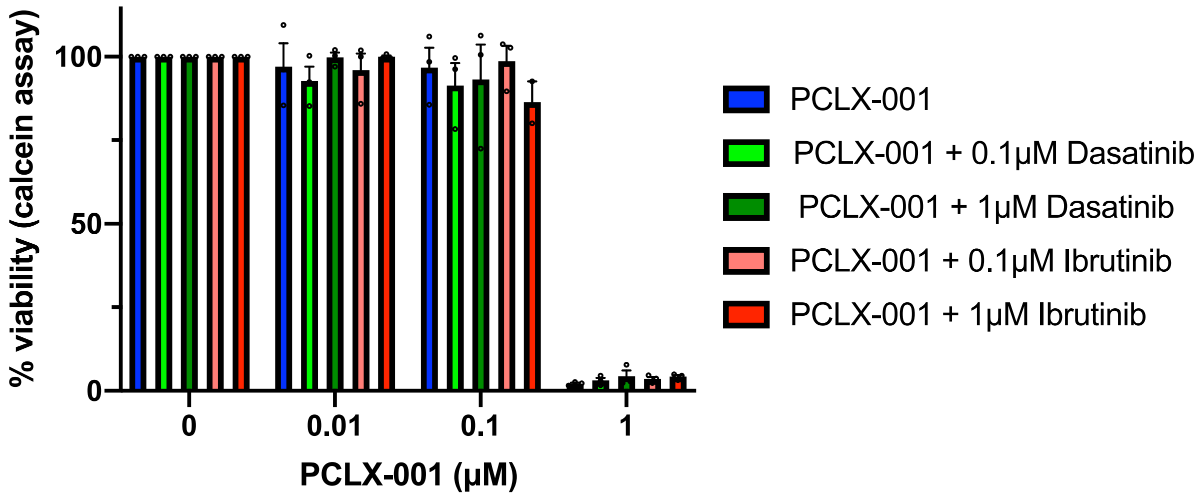

B.

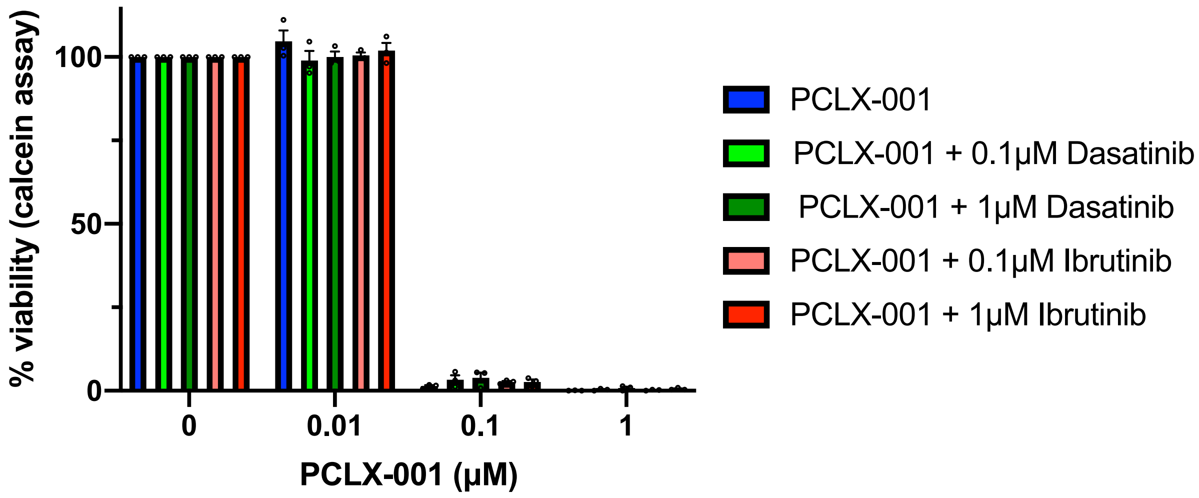

Supplementary Figure 13. Dasatinib and ibrutinib do not synergize the cytotoxic effects of PCLX-001 in IM9 and BL2 cells. IM9 (A) and BL2 (B) cells were incubated with 0.01, 0.1 and 1μM PCLX-001 in combination with 0.1 and 1 μM dasatinib or ibrutinib for 96 hours. No additive or synergistic effects were observed upon the addition of dasatinib or ibrutinin to PCLX-001. As seen throughout our experiments, malignant BL2 cells are more sensitive to PCLX-001 than normal IM9 B cells. Cell viability was measured using calcein assay and represents an average of three independent experiments. Errors bars depict s.e.m.

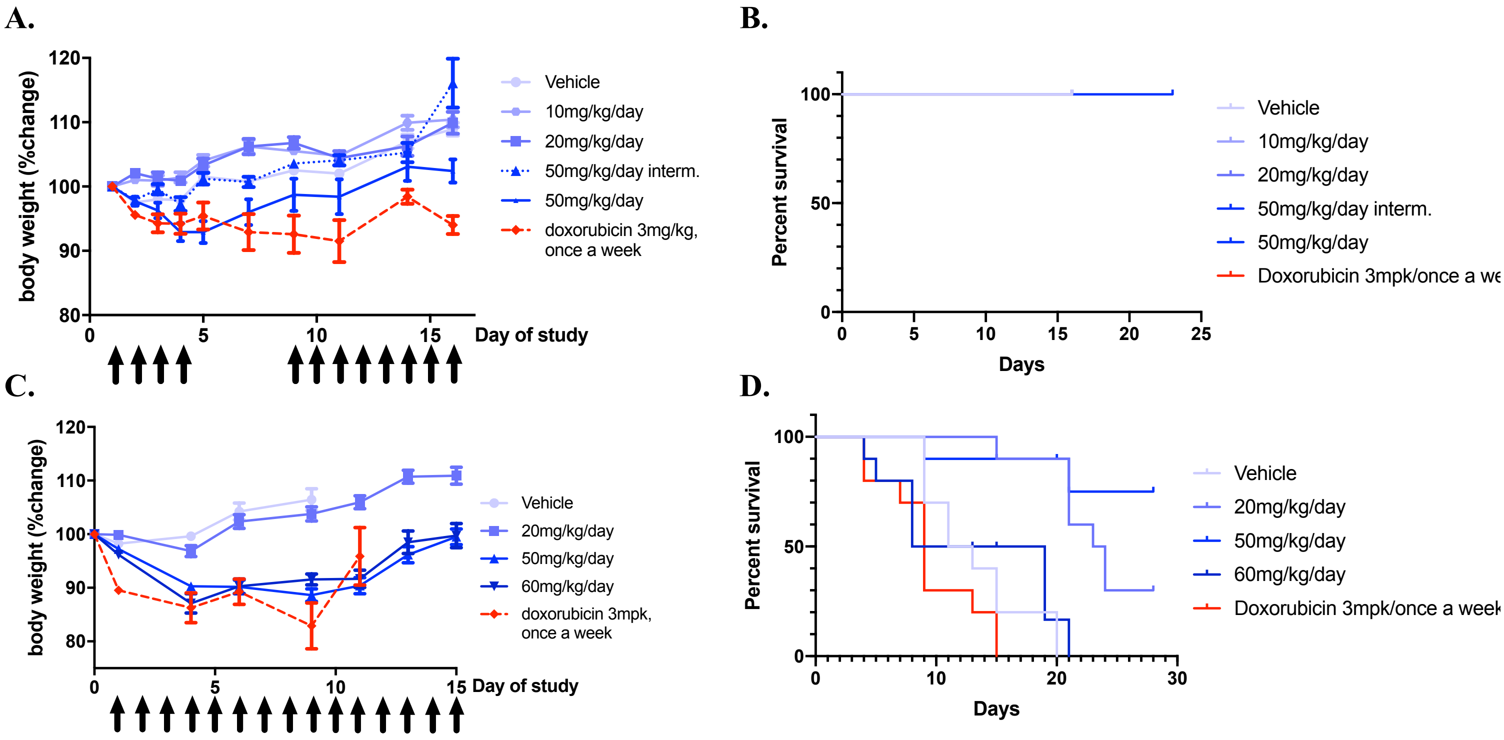

E.

Drug	Dose	Median Survival (days)
Vehicle	---	12.0
PCLX-001	20mg/kg/day	23.5
	50mg/kg/day	Not Reached
	60mg/kg/day	13.5
Doxorubicin	3mg/kg/once a week	9.0

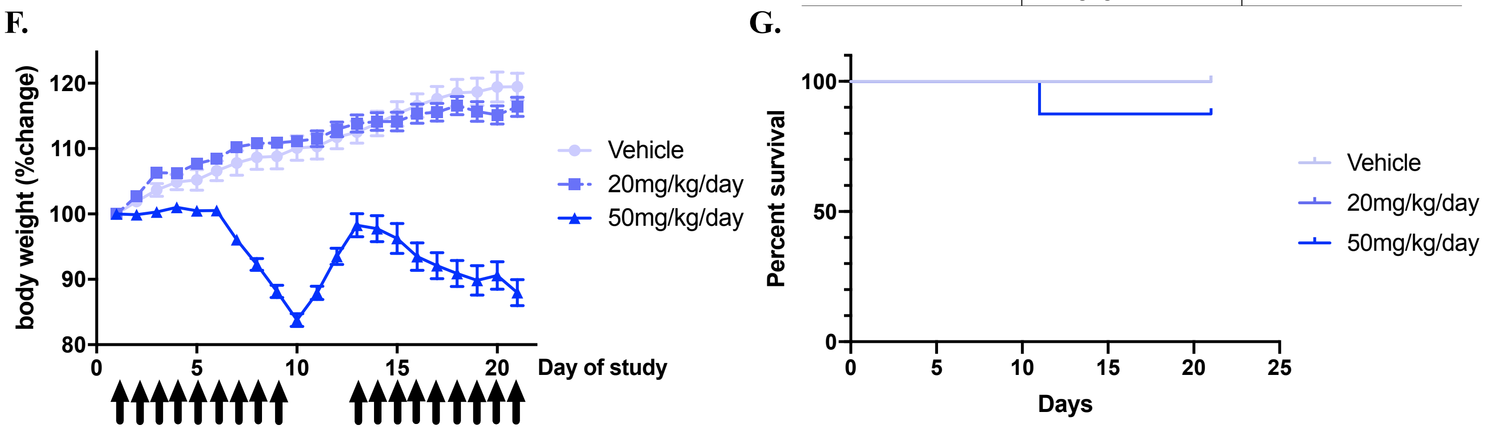

Supplementary Figure 14. Influence of PCLX-001 and doxorubicin treatment on body weight and percentage survival in xenograft models. Percentage change in body weight in DOHH2 (A), BL2 (C), and (F) DLBCL3-patient derived xenograft models (n=10/group, n=10/group and n=8/group respectively). Black arrows represent injections. Error bars represent the standard deviation in the average weight per mouse at each time point. Kaplan-Meier curves, where survival events include death from toxicity, death from cancer, or euthanasia for toxicity, depicting percent survival over time in (B) DOHH2, (D) BL2, and (G) DLBCL3-patient derived xenograft models. (E) Median survival estimates derived from Kaplan-Meier curve analysis of BL2 xenograft animals (D) following treatment with the indicated dosages of PCLX-001 and doxorubicin.

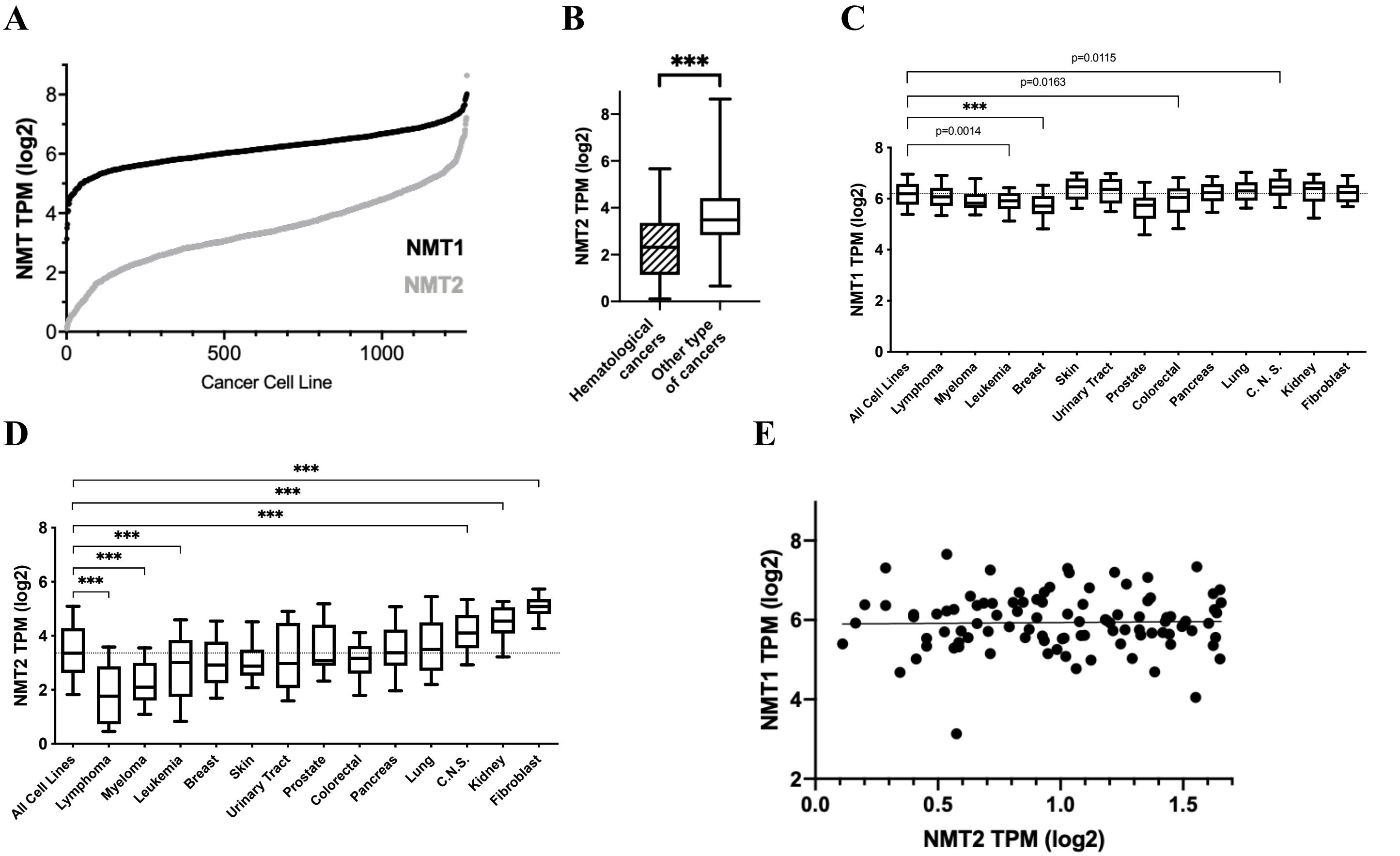

Supplementary Figure 15. ***NMT*** **expression is decreased in hematological cancer cell lines.** The average number of *NMT1*transcripts is larger than *NMT2* transcripts. However, *NMT2* transcript numbers (*grey*) show larger variations than *NMT1* transcript numbers (*black*) in cancer cell lines (A). *NMT2* mRNA expression is significantly lower in hematological cancer cell lines (Unpaired t-test; *** $P < 0.0001$) in comparison to cell lines originating from other types of cancers (Min to Max Box Plot, B). Expression of *NMT1* (C) is relatively constant across the 1269 cell lines investigated with a slight but significant decrease in expression in breast and leukemia cancer cell lines while *NMT2* expression (D) varies significantly amongst various cancers and also within a given cancer type. The data also illustrate that while the expression of *NMT2* is higher in cancer cell lines of CNS, kidney and fibroblast origins there is a selective and significant reduction of *NMT2* expression in hematological cancers such as leukemia, lymphoma and myeloma. Box plots are showing 10-90 percentiles (Ordinary one-way ANOVA, Dunnett's multiple comparisons test, *** $P < 0.0001$). *NMT1* expression is not increased in the 100 cells lines expressing the least *NMT2* as a possible compensatory mechanism (E). All data were extracted from 20Q1 PublicRNA-sequencing (Broad Institute, 1269 cell lines) and sorted in a selection of cancers.

Supplementary Table 1. Structures and basic comparison of DDD85646 and DDD86481 (PCLX-001) NMT inhibitors.

		**DDD85646**	**DDD86481** (PCLX-001)
Structure		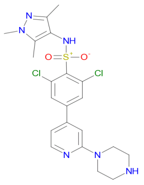	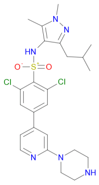
Human NMT IC_{50}		4nM	< 1 nM
Intrinsic hepat-ic clearance	Mouse (mL/min/g)	0.6	<0.5
	Rat (mL/min/g)	0.5	1.0
	Human (mL/min/g)	1.2	0.7
Fraction of drug unbound to plasma proteins (mouse/human)		0.110 / 0.176	0.067 (mouse)
Mouse IV	Clearance (mL/min/kg)	5 [3-7]	3 [2-3]
	Volume of drug distribution at steady state (L/kg)	0.6 [0.4-0.7]	0.4 [0.3-0.4]
	$T_{1/2}$ (hours)	1.3 [1.3-1.4]	1.5 [1.0-2.1]
Mouse PO 10mg/kg	Cmax (ng/mL)	2686 [2122-3755]	11201 [6986-13416]
	Tmax (hours)	0.25 [0.25-2]	2
	$T_{1/2}$ (hours)	1.2 [1.0-1.4]	5.7 [2.7-8]
	Orally absorbed drug (%)	20 [11-32]	93 [51-100]
Blood:Brain ratio		0.08	0.04

IV = intravenous; PO = Per os; Cmax = peak concentration of drug; Tmax = time to peak concentration of drug; $T_{1/2}$ – elimination half-life of drug;

Supplementary Table 2. Description of tumor and patient DLBCL3 used in murine patient-derived tumor xenograft study.

	**DLBCL3**
**Age at PDX tissue harvest**	*REMOVED
**Gender**	*REMOVED
**Clinical presentation**	Previous diagnosis of DLBCL, now presenting with pleural, bone marrow and leptomeningeal involvement
**Diagnosis**	Diffuse large B-cell lymphoma
**Cell of origin**	ABC
**Immunophenotype**	CD20+, CD10-, bcl6+, MUM1+, FoxP1+, Ki67 70-80%, cyclinD1-, c-myc 25%, bcl2+
**EBER**	Negative
**Genetic alterations**	Rearrangements of BCL6 and IGH

*REMOVED: Age and gender of the patient have been removed as possible identifiers to protect patient privacy.

Supplementary Table 3. Influence of PCLX-001 and doxorubicin treatment on serum chemistry values in DOHH2 NODscid mouse xenograft model (Supplementary Note 1). Measurements were averaged by treatment group (n=3). Standard error of the mean was calculated (SEM).

**Aspartate Transaminase U/L**	94.33 ± 26.21	104.33± 27.34	132.67 ± 55.29	125.67 ± 68.18	279.7 ± 34.17	91 ± 18.04
**Creatine PhosphoKinase U/L**	119 ± 79.7	81.7 ± 25.1	54 ± 14.7	92.3 ± 29.5	266.67 ± 69.9	80.7 ± 45.3
**Creatinine mg/dL**	0.2 ± 0.033	0.3 ± 0.067	0.3 ± 0	0.3 ± 0	0.2 ± 0	0.2 ± 0
**Bilirubin (mg/dL)**	0.1 ± 0.03	0.1 ± 0	0.1 ± 0	0.1 ± 0	0.1 ± 0	0.2 ± 0.03

Supplementary Table 4. Influence of PCLX-001 and doxorubicin treatment on hematology values in DOHH2 NODscid mouse xenograft model (Supplementary Note 1). Average measurements by treatment group (n=3). Standard error of the mean was calculated (SEM). [Normal Range]; Blue for low, Black for normal range, Red for high.

	Vehicle	10mg/kg/day	20mg/kg/day	50mg/kg/day Interm.	50mg/kg/ day	Doxorubicin 3mg/kg Once a week
**WBC (K/ul) [1.8 -10.7]**	4.38 ± 0.81	6.3± 0.12	5.41 ± 1.05	5.94 ± 1.19	1.89 ± 0.31	8.67 ± 1.58
**Absolute Neutrophil cells (K/ul) [0.1 – 2.4]**	2.9 ± 0.43	4.89 ± 0.34	3.94 ± 0.94	3.47 ± 0.65	1.45 ± 0.22	6.86 ± 1.13
**Absolute Lymphocyte cells (K/ul) [0.9 – 9.3]**	0.89 ± 0.27	0.83 ± 0.19	0.84 ± 0.15	1.51 ± 0.47	0.23 ± 0.06	0.82 ± 0.17
**Absolute Monocyte cells (K/ul) [0 -0.4]**	0.52 ± 0.13	0.52 ± 0.06	0.61 ± 0.09	0.91 ± 0.14	0.19 ± 0.06	0.95 ± 0.32
**Absolute Eosinophil cells (K/ul) [0 – 0.2]**	0.05 ± 0.02	0.05 ± 0.02	0.02 ± 0.01	0.05 ± 0.03	0.01 ± 0.01	0.01 ± 0
**Absolute Basophil cells (K/ul) [0 – 0.2]**	0.01 ± 0.00	0.01 ± 0	0.01 ± 0	0 ± 0	0 ± 0	0.03 ± 0
**RBC (M/ul) [6.36 -9.42]**	9.62 ± 0.2	9.45 ± 0.1	9.03 ± 0.32	8.07 ± 0.12	9.3 ± 0.17	8.24 ± 0.35
**Hemoglobin (g/dL) [11 – 15.1]**	14.83 ± 0.48	14.13 ± 0.18	13.97 ± 0.44	12.6 ± 0.15	14.77 ± 0.23	12.73 ± 0.41
**Hematocrit % [35.1 – 45.4]**	44.8 ± 1.52	43.2 ± 0.61	42.33 ± 1.48	39.53 ± 0.84	43.3 ± 0.7	38.23 ± 1.97
**MCV (fL) [45.4 – 60.3]**	46.57 ± 0.69	45.7 ± 0.2	46.9 ± 0.65	49.07 ± 1.72	46.6 ± 0.2	46.36 ± 0.61
**MCH (pg) [14.1 -19.3]**	15.4 ± 0.2	14.93 ± 0.03	15.43 ± 0.3	15.63 ± 0.12	15.9 ± 0.1	15.47 ± 0.23
**MCHC (g/dL) [30.2 -34.2]**	33.13 ± 0.12	32.73 ± 0.07	33.03 ± 0.22	31.90 ± 1.01	34.1 ± 0.1	33.37 ± 0.92
**RDW % [12.4 -27]**	25.63 ± 0.68	25.97 ± 0.46	25.13 ± 0.65	27.2 ± 1.82	25.8 ± 0.3	32.83 ± 0.87
**Platelets (K/uL) [592 -2972]**	1218.7 ± 211.27	1490 ± 91.31	1234.7 ± 38.49	1673.7 ± 309.81	900.7 ± 60.8	2210.7 ± 297.9
**MPV (fL) [5 – 20]**	7.23 ± 0.2	6.77 ± 0.18	7.63 ± 0.23	7.17 ± 0.09	7 ± 0.3	6.2 ± 0.15
**Reticulocyte %**	6.82 ± 1.86	8.08 ±1.58	7.21 ± 0.49	13.28 ± 6.74	6.7 ± 0.3	15.04 ± 4.87

Supplementary Note 1: Toxicology summary of the DOHH-2 NODscid xenograft (Charles River).

Design: One group of mice were given vehicle and four groups were given PCLX-001 using the dose levels and dose regimens shown in the table below.

Group no.	Group name	Dose level (mg/kg) Free base equivalent	Regimen
1	Vehicle control	0	Daily for 16 days
2	Low-dose PCLX-001	10	Daily for 16 days
4	Mid-dose PCLX-001	20	Daily for 16 days
5	High-dose PCLX-001	50	Every other day for 16 days (8 doses)
6	High-dose PCLX-001	50	Daily for 4 days, then 5-day holiday, then daily for 14 days

Mice were observed daily for clinical signs of toxicity and effects on body weight. After the last dose, three mice/group were euthanized and necropsied. At euthanasia, blood samples were taken for hematology analyses and to measure AST and CK activities and bilirubin and creatinine concentrations. At necropsy, samples of samples of femur, both kidneys, liver, small intestine, and injection site were taken and fixed. These were processed and examined microscopically by pathologist Dr. Wei-feng Dong.

Supplementary observations: The only adverse findings potentially related to PCLX-001 were in the groups given PCLX-001 at 50 mg/kg (Groups 5 and 6). With PCLX-001 every other day, RBC counts were lower than normal in all three mice, and reticulocyte and platelet counts were higher than normal in one of them. With PCLX-001 daily, neutrophil and monocyte counts were lower than normal in all three mice, and monocyte and platelet counts were lower than normal in one of them. There were no histopathologic findings in the femoral bone marrow of any of these mice.

These data are summarized in the table below.

Group no.	Group name	Dose level (mg/kg) Free base equivalent	Effect on mean body weight	Noteworthy findings after last dose
1	Vehicle **control**	0	No change for 7 days, then ↑	None
2	Low-dose PCLX-001	10	No change for 4 days, then ↑	None
4	Mid-dose PCLX-001	20	No change for 4 days, then ↑	None
5	High-dose PCLX-001	50	No change for 7 days, then ↑	↓RBCs in all three mice (low RBC count, hemoglobin, hematocrit), with ↑reticulocyte count (and platelet count) in one mouse
6	High-dose PCLX-001	50	↓ for 4 days, then ↑during holiday, then continue to ↑	↓neutrophils, ↓lymphocytes in all three mice, with ↓monocytes, ↓platelets in one mouse

At the end of the dosing period, serum AST and CK activities were higher-than-normal in one or more mice in each group, including the vehicle control group.

Supplementary Discussion/Conclusions: It is not unusual for mice to sustain some muscle damage (bruising) or liver damage from the handling required to restrain them – for example, to measure tumor size – and this can lead to increased serum AST and/or CK activity. The hematology findings in mice given PCLX-001 at 50 mg/kg were relatively mild and may reflect hematopoietic toxicity, which has been seen in rats and dogs given PCLX-001 at high dose levels[55].

Supplementary Table 5. Influence of PCLX-001 and doxorubicin treatment on average organ weight in BL2 NODscid mouse xenograft model (Supplementary Note 2). Measurements were averaged by treatment group (n=2).

	Vehicle	20mg/kg/day	50mg/kg/day	60mg/kg/day	Doxorubicin 3mg/kg Once a week
**Liver (g)**	0.22	0.23	0.18	0.23	0.20
**Kidney (g)**	0.97	1.06	1.03	0.91	0.86
**Small Intestine (g)**	1.00	0.85	0.90	0.79	0.80

Supplementary Table 6. Influence of PCLX-001 and doxorubicin treatment on serum chemistry values in BL2 NODscid mouse xenograft model (Supplementary Note 2). Measurements were averaged by treatment group (n=3). Standard error of the mean was calculated (SEM).

	Vehicle	20mg/kg/day	50mg/kg/day	60mg/kg/day	Doxorubicin 3mg/kg Once a week
**Alanine Transaminase U/L**	126.1 ± 44.8	38.6 ± 8.58	73.2 ± 32.64	137.03 ± 45.91	420.37 ± 63.74
**Aspartate Transaminase U/L**	631.2 ± 401.7	99.27 ± 20.28	170.2 ± 58.94	512.63 ± 267.45	492.23 ± 66.47
**Creatine Kinase U/L**	5304.3 ± 4689.8	124 ± 21.39	323.33 ± 87.82	3311.3 ± 2779.5	1128 ± 191.31
**Blood Urea Nitrogen mg/dL**	15.5 ± 0.5	9.03 ± 1.55	13.7 ± 2.26	10 ± 1.15	24.16 ± 3.61
**Creatinine mg/dL**	0.2 ± 0	0.13 ± 0.01	0.17 ± 0.03	0.15 ± 0.01	0.21 ± 0.04

Supplementary Table 7. Influence of PCLX-001 and doxorubicin treatment on hematology values in BL2 NODscid mouse xenograft model (Supplementary Note 2). Average measurements by treatment group (n=3). Standard error of the mean was calculated (SEM). [Normal Range]; Blue for low, Black for normal range, Red for high.

	Vehicle	20mg/kg/day	50mg/kg/day	60mg/kg/day	Doxorubicin 3mg/kg Once a week
**WBC (K/ul) [1.8 -10.7]**	4.37 ± 1.07	4.51 ± 1.44	2.89 ± 0.33	3.43 ± 0.98	4.07 ± 0.61
**Absolute Neutrophil cells (K/ul) [0.1 – 2.4]**	1.63 ± 0.41	3.15 ± 1.11	1.95 ± 0.25	1.44 ± 0.3	0.99 ± 0.13
**Absolute Lymphocyte cells (K/ul) [0.9 – 9.3]**	1.86 ± 0.87	0.68 ± 0.19	0.56 ± 0.14	1.42 ± 0.64	2.28 ± 0.4
**Absolute Monocyte cells (K/ul) [0 -0.4]**	0.81 ± 0.1	0.47 ± 0.15	0.26 ± 0.06	0.46 ± 0.2	0.7 ± 0.19
**Absolute Eosinophil cells (K/ul) [0 – 0.2]**	0.06 ± 0.02	0.2 ± 0.08	0.11 ± 0.05	0.09 ± 0.05	0.08 ± 0.03
**Absolute Basophil cells (K/ul) [0 – 0.2]**	0.01 ± 0.01	0.02 ± 0.01	0.01 ± 0.01	0.01 ± 0.01	0.02 ± 0.01
**Neutrophil % [6.6 -38.9]**	38.47 ± 8.66	69.42 ± 7.62	67.3 ± 1.46	46.44 ± 12.37	25.27 ± 3.69
**Lymphocyte % [55.8 – 91.6]**	39.76 ± 9.88	15.74 ± 3.79	19.15 ± 2.71	37.17 ± 10.76	55.48 ± 1.96
**Monocyte % [0 – 7.5]**	19.59 ± 2.27	10.41 ± 2.55	9.34 ± 2.49	12.39 ± 4.62	16.61 ± 2.68
**Eosinophil % [0 – 3.9]**	1.90 ± 0.89	4.17 ± 1.38	3.88 ± 1.62	3.52 ± 2.49	2.18 ± 0.86
**Basophil % [0 – 2]**	0.28 ± 0.17	0.27 ± 0.11	0.33 ± 0.16	0.48 ± 0.39	0.37 ± 0.15
**RBC (M/ul) [6.36 -9.42]**	10.76 ± 0.66	8.18 ± 0.28	8.94 ± 0.81	9.86 ± 0.17	10.01 ± 0.13
**Hemoglobin (g/dL) [11 – 15.1]**	14.23 ± 0.81	11.77 ± 0.68	12.4 ± 1	13.17 ± 0.65	13.6 ± 0.21
**Hematocrit % [35.1 – 45.4]**	49.67 ± 3.06	36.9 ± 1.02	39.07 ± 3.3	46 ± 0.49	45.63 ± 0.17
**MCV (fL) [45.4 – 60.3]**	46.17 ± 0.24	45.1 ± 0.35	43.73 ± 0.47	46.67 ± 0.84	45.6 ± 0.5
**MCH (pg) [14.1 -19.3]**	13.23 ± 0.09	14.4 ± 0.49	13.9 ± 0.15	13.33 ± 0.43	13.6 ± 0.12
**MCHC (g/dL) [30.2 -34.2]**	28.67 ± 0.37	31.83 ± 1.1	31.77 ± 0.19	28.63 ± 1.29	29.8 ± 0.46
**RDW % [12.4 -27]**	18.3 ± 0.25	20.63 ± 0.26	20.93 ± 0.32	22.73 ± 0.03	17.03 ± 0.15
**Platelets (K/uL) [592 -2972]**	534 ± 148.85	1188.3 ± 158.16	985.7 ± 206.8	1136.33 ± 246	975.67 ± 44.91
**MPV (fL) [5 – 20]**	5.47 ± 0.13	5.8 ± 0.06	5.9 ± 0.45	5.77 ± 0.18	5.7 ± 0.1

Supplementary Note 2: Toxicology summary of the BL2 NODscid xenograft (Jackson Lab, JAX)

Design: One group of mice were given vehicle and three groups were given PCLX-001 using the dose levels and dose regimens shown in the table below.

Group no.	Group name	Dose level (mg/kg)	Regimen
1	Vehicle control	0	Daily for 21 days
2	Low-dose PCLX-001	20	Daily for 26 days
6	Mid-dose PCLX-001	50	Daily for 21 days
7	High-dose PCLX-001	60	Daily for 21 days

Data collected were the same as in the Charles River xenograft study – clinical signs, body weight, tumor volume, blood samples from 3 mice/group for hematology and clinical chemistry (ALT, AST, BUN, creatinine, CK), and tissue samples collected and fixed from the same 3 mice. Liver, kidneys, and small intestine also were weighed.

Supplementary observations: Adverse findings potentially related to PCLX-001 were:

- Signs of ill health (e.g., rough and scruffy coats, piloerection) in most mice in groups given PCLX-001. These signs developed earlier at 50 or 60 mg/kg/day than at 20 mg/kg/day.
- Dehydration and weight loss in groups given PCLX-001 at 50 or 60 mg/kg/day. Weight loss seems to have stopped after about a week, despite continued dosing, after which mice started to gain weight.

Supplementary Discussion/Conclusions: There were no clinical pathology or anatomic pathology findings related to PCLX-001. There was a trend toward higher neutrophil counts and lower RBC counts with PCLX-001 at 20 mg/kg/day; however, this was unrelated to dose level and so was likely due to chance. Greater mean CK (and to a lesser extent, AST and ALT) activity were seen in one mouse each in Group 1 (control) and Group 4. This pattern of increase in enzyme activities strongly suggests skeletal muscle injury, which was unrelated to PCLX-001.

Supplementary Table 8. Influence of PCLX-001 and doxorubicin treatment on hematology values in DLBCL NODscid mouse patient derived xenograft model (Supplementary Note 3) Measurements were averaged by treatment group (n=3). Standard error of the mean was calculated (SEM). [Normal Range]; Blue for low, Black for normal range, Red for high.

	**Vehicle**	**20mg/kg/day**	**50mg/kg/day**
**WBC (K/ul)<br>[1.8 -10.7]**	2.11 ± 0.19	1.49 ± 0.24	2.17 ± 0.5
**Absolute Neutrophil cells (K/ul)<br>[0.1 – 2.4]**	1.39 ± 0.13	0.86 ± 0.22	1.28 ± 0.34
**Absolute Lymphocyte cells (K/ul)<br>[0.9 – 9.3]**	0.41 ± 0.07	0.4 ± 0.08	0.52 ± 0.12
**Absolute Monocyte cells (K/ul)<br>[0 -0.4]**	0.28 ± 0.03	0.23 ± 0.06	0.37 ± 0.07
**Absolute Eosinophil cells (K/ul)<br>[0 – 0.2]**	0.01 ± 0.01	0 ± 0	0 ± 0
**Absolute Basophil cells (K/ul)<br>[0 – 0.2]**	0 ± 0	0 ± 0	0 ± 0
**Neutrophil %<br>[6.6 -38.9]**	66.26 ± 0.72	57.17 ± 9.40	58.04 ± 3.38
**Lymphocyte %<br>[55.8 – 91.6]**	18.97 ± 1.96	26.99 ± 5.16	24.36 ± 2.93
**Monocyte %<br>[0 – 7.5]**	14.01 ± 2.69	15.48 ± 4.35	17.24 ± 0.68
**Eosinophil %<br>[0 – 3.9]**	0.58 ± 0.24	0.26 ± 0.08	0.32 ± 0.1
**Basophil %<br>[0 – 2]**	0.18 ± 0.05	0.1 ± 0.04	0.04 ± 0.04
**RBC (M/ul)<br>[6.36 -9.42]**	8.86 ± 0.18	8.81 ± 0.15	8.36 ± 0.83
**Hemoglobin (g/dL)<br>[11 – 15.1]**	10.9 ± 0.15	11.07 ± 0.27	10.4 ± 0.9
**Hematocrit %<br>[35.1 – 45.4]**	46.8 ± 1.16	47.13 ± 1.1	42.47 ± 4.04
**MCV (fL)<br>[45.4 – 60.3]**	52.83 ± 0.34	53.5 ± 0.36	50.83 ± 0.35
**MCH (pg)<br>[14.1 -19.3]**	12.3 ± 0.1	12.57 ± 0.19	12.5 ± 0.15
**MCHC (g/dL)<br>[30.2 -34.2]**	23.3 ± 0.29	23.50 ± 0.38	24.53 ± 0.24
**RDW %<br>[12.4 -27]**	17.7 ± 0.46	18.07 ± 0.03	20.37 ± 0.37
**Platelets (K/uL)<br>[592 -2972]**	780.67 ± 71.52	1202.33 ± 76.05	859 ± 322.66
**MPV (fL)<br>[5 – 20]**	4.97 ± 0.09	4.93 ± 0.03	5.27 ± 0.18

Supplementary Note 3: Toxicology summary of the DLBCL3 patient derived NODscid mouse xenograft:

Design: One group of mice were given vehicle and two groups were given PCLX-001 using the dose levels and dose regimens shown in the table below.

Group no.	Group name	Dose level (mg/kg)	Regimen
1	Saline control	0	Daily for 21 days
3	Low-dose PCLX-001	20	Daily for 21 days
5	High-dose PCLX-001	50	Daily for 21 days

Data collected were the same as in the previous two studies – clinical signs, body weight, tumor volume, blood samples from 3 mice/group for hematology and clinical chemistry (AST, CK, bilirubin, creatinine), and tissue samples collected and fixed from the same 3 mice.

Supplementary observations: There were no clinical signs of toxicity, effects on clinical pathology parameters, or anatomic pathology findings related to PCLX-001.

Supplementary Discussion/Conclusions: The absence of adverse effects is somewhat surprising, since it looked like there were effects on hematology parameters at 50 mg/kg/day in the study using DoHH-2 cells. Why is there a difference here is not known. Why mice tolerated daily doses at 50 mg/kg for 3 weeks in this study but not in all studies is not known. Differences including NODscid clones, chow type or microbiota might account for this.

Supplementary Table 9. List of cell lines used in this study

Cell line	Histology	Site Primary	Hist. Subtype1
**BL2**	B lymphocyte	haematopoietic and lymphoid tissue	Burkitt lymphoma
**COS-7**	fibroblast	kidney	
**DOHH2**	lymphoid neoplasm	hematopoietic and lymphoid tissue	Diffuse large B cell lymphoma
**IM9**	B lymphoblast	hematopoietic and lymphoid tissue	
**Ramos**	B lymphocyte	hematopoietic and lymphoid tissue	Burkitt lymphoma
**WSU-DLCL2**	lymphoid neoplasm	hematopoietic and lymphoid tissue	Diffuse large B cell lymphoma
**VDS**	B lymphoblast	hematopoietic and lymphoid tissue	
**BJAB**	B lymphocyte	hematopoietic and lymphoid tissue	Burkitt lymphoma
**SU-DHL-10**	lymphocyte	hematopoietic and lymphoid tissue	Diffuse large B cell lymphoma
