# Supplementary material for: Targeting N-myristoylation for therapy of B-cell lymphomas
Source: Nat Commun. 2020 Oct 22;11:5348. doi: 10.1038/s41467-020-18998-1 (PMC7582192; doi:10.1038/s41467-020-18998-1)
Supplement: Supplementary file 3 — Reporting Summary [file 41467_2020_18998_MOESM3_ESM.pdf]

nature research

Corresponding author(s): Luc G. Berthiaume

Last updated by author(s): Aug 13, 2020

Reporting Summary

Nature Research wishes to improve the reproducibility of the work that we publish. This form provides structure for consistency and transparency in reporting. For further information on Nature Research policies, see our Editorial Policies and the Editorial Policy Checklist.

Statistics

For all statistical analyses, confirm that the following items are present in the figure legend, table legend, main text, or Methods section.

n/a	Confirmed	
☐	☒	The exact sample size (n) for each experimental group/condition, given as a discrete number and unit of measurement
☐	☒	A statement on whether measurements were taken from distinct samples or whether the same sample was measured repeatedly
☐	☒	The statistical test(s) used AND whether they are one- or two-sided<br>*Only common tests should be described solely by name; describe more complex techniques in the Methods section.*
☐	☒	A description of all covariates tested
☒	☐	A description of any assumptions or corrections, such as tests of normality and adjustment for multiple comparisons
☐	☒	A full description of the statistical parameters including central tendency (e.g. means) or other basic estimates (e.g. regression coefficient) AND variation (e.g. standard deviation) or associated estimates of uncertainty (e.g. confidence intervals)
☐	☒	For null hypothesis testing, the test statistic (e.g. F, t, r) with confidence intervals, effect sizes, degrees of freedom and P value noted<br>*Give P values as exact values whenever suitable.*
☒	☐	For Bayesian analysis, information on the choice of priors and Markov chain Monte Carlo settings
☒	☐	For hierarchical and complex designs, identification of the appropriate level for tests and full reporting of outcomes
☒	☐	Estimates of effect sizes (e.g. Cohen's d, Pearson's r), indicating how they were calculated

Our web collection on statistics for biologists contains articles on many of the points above.

Software and code

Policy information about availability of computer code

Data collection	NMT1 and NMT2 mRNA expression data were extracted on March 26th 2020 from the Broad Institute CCLE database1 (https://portals.broadinstitute.org/ccle) and contained the mRNA expression data for 1269 cancer cell lines. The RNAseq TPM gene expression data (Expression Public 20Q1) were analysed for protein coding genes using RSEM and are presented as Log2 transformed values using a pseudo-count of 1.
Data analysis	Cell count was performed using a Cytation 5 Cell Imaging Multi-Mode Reader (Biotek Instruments, Inc.) and analysed by Biotek Gen5 Data Analysis software (2.09).<br>All data points for the Horizon robotic analysis are collected via automated processes and are subject to quality control and analyzed using Horizon's Chalice Analyzer proprietary software (1.5).<br>EC50 for the ChemPartner Robotic analysis was calculated using XLfit software (5.5).<br>Images were acquired using a Zeiss Observer Z1 microscope and Axiovision software (Axiovision, version 4.8).<br>Data were analyzed using Prism 8 software (GraphPad) (8.4.1)

For manuscripts utilizing custom algorithms or software that are central to the research but not yet described in published literature, software must be made available to editors and reviewers. We strongly encourage code deposition in a community repository (e.g. GitHub). See the Nature Research guidelines for submitting code & software for further information.

Data

Policy information about availability of data

All manuscripts must include a data availability statement. This statement should provide the following information, where applicable:

- Accession codes, unique identifiers, or web links for publicly available datasets
- A list of figures that have associated raw data
- A description of any restrictions on data availability

The data that support the findings of this study are available from the corresponding author upon reasonable request.

Field-specific reporting

Please select the one below that is the best fit for your research. If you are not sure, read the appropriate sections before making your selection.

[x] Life sciences [ ] Behavioural & social sciences [ ] Ecological, evolutionary & environmental sciences

For a reference copy of the document with all sections, see nature.com/documents/nr-reporting-summary-flat.pdf

Life sciences study design

All studies must disclose on these points even when the disclosure is negative.

Sample size	Groups of 8 to 10 animals were used for all animal studies. No sample-size calculation was performed. 8 animals per group were sufficient to show a significant effect of our treatment and minimal standard deviation. Larger groups would have been an unnecessary waste of resources, funding and animals.
Data exclusions	No Data exclusion
Replication	All experiments were successfully replicated at least 3 times.
Randomization	All samples/animals were randomly allocated into experimental groups.
Blinding	Investigators were blinded during robotic cell line screens and animal xenograft studies as they were performed and analysed by 3rd party research companies (Horizon (St. Louis, MO) ; OncolinesTM (Oss, Netherlands) ; (Chempartner, Shanghai, China) ; Charles River , Jackson Laboratory and/or collaborators at the Singapore General Hospital's facilities (SGH)).

Reporting for specific materials, systems and methods

We require information from authors about some types of materials, experimental systems and methods used in many studies. Here, indicate whether each material, system or method listed is relevant to your study. If you are not sure if a list item applies to your research, read the appropriate section before selecting a response.

Materials & experimental systems

n/a	Involved in the study	
[ ]	[x]	Antibodies
[ ]	[x]	Eukaryotic cell lines
[x]	[ ]	Palaeontology and archaeology
[ ]	[x]	Animals and other organisms
[ ]	[x]	Human research participants
[x]	[ ]	Clinical data
[x]	[ ]	Dual use research of concern

Methods

n/a	Involved in the study	
[x]	[ ]	ChIP-seq
[x]	[ ]	Flow cytometry
[x]	[ ]	MRI-based neuroimaging

Antibodies

Antibodies used	Rabbit anti-PARP-1 (1:5000, affinity purified polyclonal#EU2005, lot 1), anti-GAPDH (1:5000, affinity purified polyclonal, #EU1000, lot 1) and anti-GFP (1:10000, affinity purified, #EU1, lot B3-1) were from laboratory stock and are available through Eusera (www.eusera.com). Our affinity purified rabbit anti-GFP is also available as Ab6556 from Abcam (Cambridge, MA). Rabbit monoclonal anti-Src (1:2000, clone 32G6, #2123, lot 5), Lyn (1:2000, clone C13F9, #2796, lot 4), P-Lyn Y507 (1:5000, polyclonal, #2731, lot 5), Fyn (1:2000, polyclonal, #4023, lot 3), Lck (1:2000, clone D88, #2984, lot 4), Hck (1:2000, clone E1I7F, #14643, lot 1), c-Myc (1:10000, clone D3N8F, #13987, lot 5), ERK (1:2000, clone 4695, #9102, lot 27), P-ERK (1:5000, clone 3510, #9101, lot 30), P-SFK (1:10000, clone D49G4, #6943, lot 4), BTK (1:2000, clone D3H5, #8547, lot 13), P-BTK Y223 (1:5000, clone D9T6H, #87141, lot 1) SYK (1:2000, clone D3Z1E, #13198, lot 5), P-SYK Y525/526 (1:5000, clone C87C1, lot 18) and anti-cleaved caspase-3 (1:1000, clone 5A1E, #9664, lot 20) were purchased from Cell Signaling Technologies. Rabbit monoclonal anti-BIP (1:2000, polyclonal, ADI-SPA-826) was purchased from Enzo Life Sciences. Rabbit anti-Mcl-1 (1:2000, clone Y37, #32087, lot GR119342-5), NFkB (1:2000, clone E379, #32536, lot

GR3199609-2), P-Lyn Y396 (1:5000, polyclonal, #226778, lot GR3195652-5) were purchased from Abcam (Cambridge, MA). Mouse monoclonal anti-p-Tyr (1:10000, PY99, sc-7020, lot I2118) antibody was purchased from Santa Cruz Biotechnology. Mouse anti human HGAL was purchased at eBioscience (1:10000, clone 1H1-A7, #14-9758-82, lot E24839-101). Rabbit polyclonal anti-ARF-1 antibody (1:2000, polyclonal, #PA1-127, lot TK 279638) was purchased from ThermoFisher Scientific.
Goat anti-Human IgM (µ chain) (70-8028-M002, lot S728028002001) was purchased from Tonbo biosciences. Goat F(ab')2 anti-human IgM was purchased from BioRad (STAR146, lot 152684). Rabbit Anti-human Src antibody from Sigma-Aldrich (polyclonal, Ab-529, lot 871521168) was used for immunoprecipitation.

Validation

All antibodies of the highest specificity based on the supplier's validation were selected for use. We confirmed by immunoblotting that the size of the target recognized by the various antibodies used in western blotting procedures corresponded to that described in the suppliers'quality control data file supplied in print or online.
abbit anti-PARP-1 (1:5000, affinity purified polyclonal#EU2005, lot 1), anti-GAPDH (1:5000, affinity purified polyclonal, #EU1000, lot 1) and anti-GFP (1:10000, affinity purified, #EU1, lot B3-1) were validated using immunoblotting on various cell lines lysates by Eusera.
Rabbit monoclonal anti-Src (1:2000, clone 32G6, #2123, lot 5) was validated by Cell Signaling Technologies by immunobloting in various cell types.
Rabbit monoclonal anti-Lyn (1:2000, clone C13F9, #2796, lot 4) was validated by Cell Signaling Technologies by immunobloting and IHC-P in various cell types.
Rabbit anti P-Lyn Y507 (1:5000, polyclonal, #2731, lot 5) was validated by Cell Signaling Technologies by immunobloting in various cell types activated by anti-IgM antibody.
Rabbit anti Fyn (1:2000, polyclonal, #4023, lot 3) was validated by Cell Signaling Technologies by immunobloting in various cell types.
Rabbit anti Lck (1:2000, clone D88, #2984, lot 4) was validated by Cell Signaling Technologies by immunobloting and IHC-P in various cell types.
Rabbit anti Hck (1:2000, clone E1I7F, #14643, lot 1) was validated by Cell Signaling Technologies by immunobloting and immunoprecipitation in various cell types.
Rabbit anti c-Myc (1:10000, clone D3N8F, #13987, lot 5) was validated by Cell Signaling Technologies by immunobloting in c-Myc(-)/ N-Myc(+) K562 cells.
Rabbit anti ERK (1:2000, clone 4695, #9102, lot 27) was validated by Cell Signaling Technologies by immunobloting in SiRNA treated HEK293 cells.
Rabbit anti P-ERK (1:5000, clone 3510, #9101, lot 30) was validated by Cell Signaling Technologies by immunobloting mouse enbryonic fibroblast treated with MEK1/2 inhibitor (U0196) and confocal immunofluorescence.
Rabbit anti P-SFK (1:10000, clone D49G4, #6943, lot 4), was validated by Cell Signaling Technologies by immunobloting in NIH/3T3 cells treated with human Paltelet-Derived Growth factor BB hPDGF-BB#8912.
Rabbit anti BTK (1:2000, clone D3H5, #8547, lot 13) was validated by Cell Signaling Technologies by immunobloting and IHC-P in various cell types.
Rabbit anti P-BTK Y223 (1:5000, clone D9T6H, #87141, lot 1) was validated by Cell Signaling Technologies by immunobloting in Ramos cells treated with anti-human IgM.
Rabbit anti SYK (1:2000, clone D3Z1E, #13198, lot 5) was validated by Cell Signaling Technologies by immunobloting, IHC-P and immunoprecipitation in various cell types.
Rabbit anti P-SYK Y525/526 (1:5000, clone C87C1, lot 18) was validated by Cell Signaling Technologies by immunobloting in Ramos cells treated with anti-human IgM, IF-IC and Flow cytometry.
Rabbit anti-cleaved caspase-3 (1:1000, clone 5A1E, #9664, lot 20) was validated by Cell Signaling Technologies by immunobloting ivarious cell lines treated with staurosporine or etoposide.
Rabbit monoclonal anti-BIP (1:2000, polyclonal, ADI-SPA-826) was validated by Enzo Life Sciences bi immunoblotting of Grp78 (BIP) recombinant protein, CHO-K1 cell lysate and mouse liver microsome tissue extract.
Rabbit anti-Mcl-1 (1:2000, clone Y37, #32087, lot GR119342-5),were validated by Abcam (Cambridge, MA) by immunoblotting in Mcl-1 KO HEK293T cells.
Rabbit anti NFkB (1:2000, clone E379, #32536, lot GR3199609-2) were validated by Abcam (Cambridge, MA) by immunoblotting in Mcl-1 KO HAP1 cells.
Rabbit anti P-Lyn Y396 (1:5000, polyclonal, #226778, lot GR3195652-5) were validated by Abcam (Cambridge, MA) by immunoblotting in K562 cells treated with calf-intestinal alkaline phosphatase.
Mouse monoclonal anti-p-Tyr (1:10000, PY99, sc-7020, lot I2118) antibody was validated by Santa Cruz Biotechnology by immunoblotting in EGF treated A-431 whole cell lysates.
Mouse anti human HGAL was validated by eBioscience (1:10000, clone 1H1-A7, #14-9758-82, lot E24839-101) by immunohistochemistry of embedded human tonsil.
Rabbit polyclonal anti-ARF-1 antibody (1:2000, polyclonal, #PA1-127, lot TK 279638) was validated by ThermoFisher Scientific by immunoblotting in ARF1 siRNA mediated knockdown in Hela cells.
Rabbit Anti-human Src antibody was validated by Sigma-Aldrich (polyclonal, Ab-529, lot 871521168) by immunobloting in various cell types.
Goat anti-Human IgM (µ chain) (70-8028-M002, lot S728028002001) was validated by Tonbo biosciences for immunoassay applications.
Goat F(ab')2 anti-human IgM was validated by BioRad (STAR146, lot 152684) for immunoassay applications.

Eukaryotic cell lines

Policy information about cell lines

Cell line source(s)

IM9, Ramos, SU-DHL-10 and COS-7 were purchased from ATCC. BL2, DOHH2, WSU-DLCL2 & BJAB were purchased from DSMZ (Germany). Ramos and BL2 were kind gifts of Drs. Jim Stone and Robert Ingham of University of Alberta. VDS isolation was described in Tosato G, et al. (reference 47). VDS, BJAB and SU-DHL-10 were kind gifts of Dr. Michael Gold of the University of British Columbia. HUVEC cells (pooled from up to 4 umbilical cords) were purchased from PromoCell. All cell lines identity was confirmed at The Genetic Analysis Facility, The Centre for Applied Genomics, The Hospital for Sick Children,

	Peter Gilgan Centre for Research and Learning, 686 Bay St., Toronto, ON, Canada M5G 0A4 (www.tcag.ca).
Authentication	All cell lines identity was confirmed by STR profiling at The Genetic Analysis Facility, The Centre for Applied Genomics, The Hospital for Sick Children, Peter Gilgan Centre for Research and Learning, 686 Bay St., Toronto, ON, Canada M5G 0A4 (www.tcag.ca)
Mycoplasma contamination	Cell lines were tested regularly for mycoplasma contamination using MycoAlert Plus Mycoplasma Detection Kit (Lonza, ME, USA). All cell lines tested negative for mycoplasma contamination.
Commonly misidentified lines (See ICLAC register)	We did not used any commonly misidentified cell lines.

Animals and other organisms

Policy information about studies involving animals; ARRIVE guidelines recommended for reporting animal research

Laboratory animals	DOHH2 xenograft study at Charles River's facility: Female severe combined immunodeficient mice (Fox Chase SCID®, C.B-17/Icr-Prkdcscid/IcrIcoCrl, Charles River) were nine weeks old on Day 1 of the study and had a BW range of 17.8–22.9 g. The animals were fed ad libitum water (reverse osmosis, 1 ppm Cl) and NIH 31 Modified and Irradiated Lab Diet® consisting of 18.0% crude protein, 5.0% crude fat, and 5.0% crude fiber. On Day 1 of the study, animals were given a rehydration solution ad libitum in an effort to reduce dehydration during the dosing phase of the study. The rehydration solution consisted of 0.45% NaCl : 2.5% glucose : and 0.075% KCl in sterile water. The mice were housed on irradiated Enrich-o'cobs™ bedding in static microisolators on a 12-hour light cycle at 20–22 °C (68–72 °F) and 40–60% humidity.<br><br>BL2 xenograft study at Jackson Laboratory: One hundred five (105) 6 week old female NOD.CB17-Prkdc scid/J (NOD scid, Stock #001303) mice were transferred to the in vivo research laboratory in Sacramento, CA. The mice were ear notched for identification and housed in individually and positively ventilated polysulfone cages with HEPA filtered air at a density of 5 mice per cage. Initially cages were changed every two weeks. The animal room was lighted entirely with artificial fluorescent lighting, with a controlled 12 hour light/dark cycle (6am to 6pm light). The normal temperature and relative humidity ranges in the animal rooms were 20-26°C and 30-70%, respectively. The animal rooms were set to have up to 15 air exchanges per hour. Filtered tap water, acidified to a pH of 2.5 to 3.0, and standard lab chow were provided ad libitum.<br><br>Mice used for the patient derived xenograft were purchased from InVivos, Singapore and fed with standard laboratory diet and distilled water ad libitum. The animals were kept on a 12 h light/dark cycle at 22 ± 2°C in BRC, A*STAR and maintained in accordance with the institutional guidelines.
Wild animals	Study did not involved wild animals
Field-collected samples	Samples did not involved field-collected samples.
Ethics oversight	Charles River Discovery Services North Carolina (CR Discovery Services) specifically complies with the recommendations of the Guide for Care and Use of Laboratory Animals with respect to restraint, husbandry, surgical procedures, feed and fluid regulation, and veterinary care. The animal care and use program at CR Discovery Services is accredited by the Association for Assessment and Accreditation of Laboratory Animal Care International, which assures compliance with accepted standards for the care and use of laboratory animals.<br>In Vivo Services at The Jackson Laboratory - Sacramento facility, an OLAW-assured and AAALAC-accredited organization conducted the DOHH2 mouse xenograft study. It was performed according to an IACUC-approved protocol and in compliance with the Guide for the Care and Use of Laboratory Animals (National Research Council, 2011).<br>For the DLBCL patient derived xenograft, the experimental protocol (#130812) was approved by the Institutional Animal Care and Use Committee (IACUC) of the Biological Resource Center (BRC), A*STAR.

Note that full information on the approval of the study protocol must also be provided in the manuscript.

Human research participants

Policy information about studies involving human research participants

Population characteristics	2 healthy human research volunteers were recruited for PBMC and lymphocytes isolation for a 20 ml blood collection (patient #1: male, 34 years old, no diagnosis, no treatment; patient #2: male, 54 years old, no diagnosis, no treatment).<br>Patient DLBCL3 was a 58 year-old male who had been treated for Stage I diffuse large B-cell lymphoma at age 43 with cyclophosphamide, doxorubicin, vincristine, and prednisolone (CHOP), which resulted in complete remission (Table S2). Patient DLBCL3 then presented to Singapore General Hospital 10 years subsequently with recurrent disease in the bone marrow and leptomeninges and pleural effusions. He received two courses of rituximab, ifosfamide, carboplatin, and etoposide and intrathecal methotrexate/cytarabine, followed by four courses of dexamethasone, cytarabine, and cisplatin and intrathecal methotrexate. His tissue was harvested for PDX propagation at this time. His disease continued to progress, and he died a year later.
Recruitment	2 healthy human research volunteers were recruited at the University of Alberta.<br>DLBCL3 patient was recruited at Singapore General Hospital and written informed consent was obtained for use of these samples for the specific research purpose only.
Ethics oversight	Study protocol was approved by the Health Research Ethics Board of Alberta Cancer Committee (Study title: Evaluations of Fatty AcylTransferases (FATs) in fresh blood and blood forming cells; HREBA.CC-17-0624).

For the DLBCL patiend derived xenograft, all procedures involving human samples were approved by and performed in accordance with the ethics principles of the Sing Health Centralized Institutional Review Board. Written informed consent was obtained for use of these samples for the specific research purpose only.

Note that full information on the approval of the study protocol must also be provided in the manuscript.
